# The role of action effects in motor sequence planning and execution: exploring the influence of temporal and spatial effect anticipation

**DOI:** 10.1007/s00426-021-01525-2

**Published:** 2021-06-29

**Authors:** Rachel M. Brown, Erik Friedgen, Iring Koch

**Affiliations:** Cognitive and Experimental Psychology, Institute of Psychology, RWTH Aachen University, Jaegerstrasse 17–19, Building 6011, Aachen, 52066 Germany

## Abstract

Actions we perform every day generate perceivable outcomes with both spatial and temporal features. According to the ideomotor principle, we plan our actions by anticipating the outcomes, but this principle does not directly address how sequential movements are influenced by different outcomes. We examined how sequential action planning is influenced by the anticipation of temporal and spatial features of action outcomes. We further explored the influence of action sequence switching. Participants performed cued sequences of button presses that generated visual effects which were either spatially compatible or incompatible with the sequences, and the spatial effects appeared after a short or long delay. The sequence cues switched or repeated across trials, and the predictability of action sequence switches was varied across groups. The results showed a delay-anticipation effect for sequential action, whereby a shorter anticipated delay between action sequences and their outcomes speeded initiation and execution of the cued action sequences. Delay anticipation was increased by predictable action switching, but it was not strongly modified by the spatial compatibility of the action outcomes. The results extend previous demonstrations of delay anticipation to the context of sequential action. The temporal delay between actions and their outcomes appears to be retrieved for sequential planning and influences both the initiation and the execution of actions.

**Supplementary Information:**

The online version contains supplementary material available at 10.1007/s00426-021-01525-2.

## Introduction

Voluntary action involves using body movements to effect perceivable changes in the environment. Because our actions are outcome-directed, the memory trace or anticipation of the outcomes may function to control the actions that produced them. This notion is articulated by the ideomotor principle (James, 1890). According to this principle, actions are preceded by anticipating their perceived consequences (e.g., Greenwald, 1970; Pfister, 2019; Shin et al., 2010). This principle has been useful for unifying a wide range of evidence for perception–action integration in the planning of actions. It is now understood that learned associations between actions and the stimuli they generate can be bi-directional: actions can prime the perception of their associated effects (or outcomes), and action effects can prime their associated actions (e.g., Land, 2018; Thomaschke et al., 2018). Importantly, the ideomotor principle holds that the second priming mechanism is necessary for action control: performing an action depends on first retrieving, or anticipating, its perceived effect (from here on we refer to action outcomes as action “effects”). In accord with this notion, ample evidence suggests that actions are facilitated when their associated effects either precede or predictably follow those actions (e.g., Elsner & Hommel, 2001; Kunde, 2001; for a review see Shin et al., 2010). Evidence suggests that effect anticipation, or the retrieval of an action’s effect prior to performing the action, can speed action selection or initiation (Koch & Kunde, 2002; Kunde, 2001, 2003; Kunde et al., 2004), as well as action execution (Kunde et al., 2004).

An open question that is not addressed by the ideomotor principle is how the anticipation of different action effects influences sequential action. Many of the actions we perform daily are sequential: they involve performing series of movements (or efferent outputs) in a specific temporal order that, with practice, become integrated into a single fluent action (Lashley, 1951; Rosenbaum et al., 1983; Stöcker & Hoffmann, 2004). Sequential actions include changing gears while driving, typing a word on the computer, or speaking a common greeting. Sequential actions also produce perceivable effects, such as words on a screen when typing, or melodies when performing music. Previous work has shown that the visual or auditory effects caused by sequential actions play a role in controlling those actions (Pfordresher, 2003; Pfordresher et al., 2011; Snyder et al., 2015). The ideomotor principle may play a role in pre-planning multi-step movements such as these. Sequential action requires first encoding and later retrieving the temporal order of efferent outputs. The binding of contingent action effects to those outputs during encoding may facilitate the retrieval of (1) the efferent outputs themselves, and (2) their temporal order. This possibility is suggested by the influence of action–effect associations (e.g., associations between key presses and contingent tones) on motor sequence learning. At least several studies have demonstrated that action–effect associations facilitate sequence learning in the context of the serial reaction time task, a task in which participants respond to visual stimuli at different spatial locations on a screen by pressing spatially-corresponding buttons. In this paradigm, participants respond faster when the visual stimuli follow a regular sequence, compared to when they appear randomly, indicating sequence learning (see for example, Brown & Robertson, 2007). When each response in the task additionally produced a contingent and unique tone, sequence learning improvements were greater compared to when responses produced no tones or unpredictable tones, even though the tones were not relevant to the task (Esser & Haider, 2018; Haider et al., 2020; Hoffmann et al., 2001; Stöcker et al., 2003). In addition, the presence of response-contingent auditory effects (such as tones) improved participants’ ability to verbally recall the sequence following learning (Esser & Haider, 2018; Haider et al., 2020; Zirngibl & Koch, 2002). The authors suggested that an ideomotor process facilitated sequence learning: the learned association between responses and their effects later allowed the effects to be utilized to retrieve the responses (Stöcker et al., 2003). This evidence raises the possibility that action effects associated with sequential efferent outputs may continue to play a role in sequential control, even after learning has taken place.

If effect anticipation facilitates sequence learning, two questions follow: (1) does effect anticipation aid action sequence retrieval following learning, and (2) what kind of action effects influence sequential action control? The first question has been addressed by a series of studies examining how the presence of contingent action effects influenced the planning of already-learned action sequences. These studies examined the influence of spatial compatibility between actions and their effects (Keller & Koch, 2006, 2008). Compatibility refers to the degree of feature overlap between the actions and effects (e.g., Greenwald, 1972). For instance, single button presses were performed faster when they were performed in the same location (left or right) as the location of the visual effect (e.g., Kunde, 2001). In a sequential task that mimicked musical performance, participants made series of button presses on three vertically-aligned buttons, each of which produced tones of varying pitch height (Keller & Koch, 2008). The pitch height of the tones was mapped to button height in either a compatible manner, where the highest button produced the highest pitch (middle button = middle pitch, lowest button = lowest pitch) or an incompatible manner, where the highest button produced the middle or lowest pitch. When button-to-pitch mapping was compatible, participants were faster to initiate the sequences of button presses, compared to when the mapping was incompatible.

The second question, what kinds of action effects influence sequential action control, remains relatively unexplored. Any given action produces a range of effects that are perceived through various sensory channels. Somatosensory action effects can include kinesthetic, proprioceptive, vestibular, or tactile feedback, and action effects can also include perceived changes in the environment such as sounds or images (Pfister, 2019). The latter types of action effects can include both spatial and temporal features: we can perceive where and when an effect occurred. Previous work using single, discrete actions (such as simple button presses) has shown that, in addition to spatial effects described above (Kunde, 2001), temporal effects can also influence action planning (Kunde, 2003). Single responses were initiated faster when response effects had a shorter duration (Kiesel & Hoffmann, 2004; Kunde, 2003). Single responses were also initiated faster when there was a short delay of 50 milliseconds (ms) between the response and the action effect onset compared to a long delay of 2 s (Dignath et al., 2014). Further work showed that this “delay anticipation” effect was not modified by the predictability of the effect identities, which were in this case the pitch or timbre of tone effects. The longer initiation times in the case of a longer action–effect delay is assumed to reflect anticipation of the temporal interval between action and effect, because longer intervals should take longer to retrieve than shorter intervals (Decety & Jeannerod, 1995; Dignath & Janczyk, 2017; Jeannerod, 2001). The evidence further suggests that the temporal interval between a response and effect can be retrieved independently from features of the effect itself (Dignath & Janczyk, 2017). Based on this evidence, it has been hypothesized that temporal and spatial features of action effects can be retrieved independently in action control (*separate trace account*; Dignath & Janczyk, 2017). It is not known whether this is the case for sequential action control.

We here extend previous work on temporal and spatial effect anticipation to sequential action. We investigate whether sequential action planning is influenced by the anticipation of temporal or spatial features of action effects. Some previous work has shown that the ability to entrain sequential actions to temporally-regular cues is influenced by spatially-compatible action effects (Keller & Koch, 2006). However, we do not yet know how temporal variations of action effects, particularly in the presence of additional spatial action effects, influence sequential action control. In particular, it is not yet known whether sequential planning is influenced by variations in the temporal delay between actions and effects. The goal of the current study was to examine whether the temporal delay between actions and effects has an influence on sequential actions even when spatial action effects are also present. In this way we aim to replicate and extend previous demonstrations of delay anticipation in action planning.

An additional unexplored question in anticipatory action control is how action planning is influenced by higher-order planning, or switching from one particular action sequence to another. Normally we perform sequential actions within a larger series of actions. When we enter a room we open the door and then turn on the light. When we drive we alternate between shifting gears and steering. Importantly, higher-order planning requires flexibility. The order in which we perform lower-level units of actions is not always predictable and must often be updated based on current circumstances. When we enter a different room we may open a window after opening the door. When we drive, external cues such as speed limit signs or traffic conditions tell us when we need to switch from steering to gear-shifting. These higher-order planning demands may influence how features of action effects are utilized in action planning. The influence of action sequence switching on ideomotor action planning is largely unknown. Decades of research have shown that action sequence switching facilitates motor sequence *learning* (Lin et al., 2013; for an early review, see Magill & Hall, 1990; Shea & Morgan, 1979), but the influence of action switching on sequence *planning* has received less attention. So far studies have addressed the influence of contextual switching on effect anticipation when planning single responses (e.g., button presses). Evidence from these studies suggests that effect anticipation can be context-specific, and can persist even when the context switches from one action to the next (Kiesel & Hoffmann, 2004). The question remains how instructed (or cued) switching of action sequences could influence the anticipation of the perceptual action effects. The current study therefore explored how switching from one cued action to another influences effect anticipation in (sequential) action control.

## Current study

The current study aimed first to extend previous work on temporal and spatial effect anticipation to sequential action, by addressing how multiple action effect features are retrieved and utilized in sequential action control. We examined whether sequential action planning is influenced by the temporal delay between actions and effects, even in the presence of spatial action effects. We therefore manipulated the temporal delay and the spatial compatibility between actions and effects. Participants performed two sequences, each consisting of pressing three keys on a computer keyboard. The sequences were initially memorized and associated with particular cues (colors) which functioned as imperative cues for the sequences. Correct sequences of key presses generated both spatial and temporal action effects. Spatial effects consisted of squares moving across the screen in either the same direction as the key presses (compatible spatial effects) or the opposite direction as the key presses (incompatible spatial effects). An advantage of this action effect assignment is that visual-spatially compatible action effects do not need to be learned; rather they exploit a natural feature overlap along the dimension of space (dimensional overlap, see Kornblum et al., 1990). Temporal effects consisted of a delay between the end of sequence production and the onset of the spatial effects. The duration of the delay was either short (50 ms) or long (1200 ms). The spatial and temporal action effect manipulations were fully crossed, yielding four possible spatial–temporal conditions (compatible-short, compatible-long, incompatible-short, incompatible-long). Each condition was blocked such that spatial compatibility and delay were consistent (and thus perfectly predictable) across consecutive trials. These manipulations addressed our first aim to examine whether sequential actions are influenced by temporal effect anticipation, even when those action effects also vary spatially.

We additionally explored how anticipatory sequential action control is influenced by cued action switching. We varied whether cued action sequences switched or repeated across trials. We did so in such a way that teased apart the repetition of the action cue (the cue signified which sequence to perform) and the repetition of the action sequence. We assigned two cues (two colors) to each sequence, which were memorized before the experiment. Cue switches could therefore involve either repeating the same action sequence or switching to a different action sequence. In addition, we varied the relevance of the action cues for action switching across two groups of participants. For Group 1, sequences followed a fixed order across trials (“Predictable Sequence” group), and for Group 2, sequences followed an unpredictable order (“Unpredictable Sequence” group). Thus, for Group 1 the cues should have been less relevant for successful switching than for Group 2, because knowledge of the upcoming sequence was provided by the fixed order of sequences across trials (even though the cues were manipulated in the same way in both groups, and the same transitions were possible across trials: both cue and sequence repeat, cue switch/sequence repeat, both cue and sequence switch). This approach varied the difficulty to retrieve correct action sequences across trials and allowed us to examine how higher-order planning influences effect anticipation in sequential action control.

We hypothesized that sequence performance would be influenced by both delay and the spatial compatibility of action effects. In accord with previous research, we expected that temporal and spatial action effects would influence sequence production independently. We expected the shorter effect delay to speed sequence initiation regardless of spatial compatibility, and we expected compatibility to speed sequence initiation regardless of delay, in accord with the “separate-trace” hypothesis of multi-feature effect anticipation. With regard to cued action sequence switching, in the absence of strong predictions from previous work, we assumed that cue or action switching could either reduce or enhance temporal or spatial effect anticipation. Switching cues or action sequences could reduce effect anticipation by increasing working memory demands, or switching could increase effect anticipation by increasing reliance on the anticipated effects as a mnemonic aid for retrieving the correct sequence.

## Method

### Participants

Participants included 40 neurologically healthy adult volunteers from the RWTH Aachen student community. Group 1 consisted of 20 participants (15 females) with a mean age of 22.4 years (SD 3.1, 18–30). Group 2 consisted of 20 participants (14 females) with a mean age of 23.4 years (SD 2.8, 18–28). All participants were right-handed, except for one left-handed participant in Group 1, and one left-handed participant in Group 2. Participants provided written informed consent, and the study was conducted according to the Declaration of Helsinki.

An a-priori power analysis was performed based on a delay-anticipation effect size of $\eta_{{\text{p}}}^{2}$ = 0.35 (Dignath & Janczyk, 2017) and a minimum spatial compatibility effect size of $\eta_{{\text{p}}}^{2}$ = 0.41 (others reported $\eta_{{\text{p}}}^{2}$ = 0.54) (Keller & Koch, 2008; Kunde, 2001). Because the ideomotor principle and the separate-trace account do not make assumptions about effect sizes, we instead based effect size estimates on previous experiments with a similar design as that of the current study: a within-subjects design with a 2-level manipulation of delay or spatial action effects. Power analysis using an alpha level corrected for the number of effects of interest, delay and compatibility (*α* = 0.05/2 = 0.025), and a power level of 1 − *β* = 0.80 suggested a sample size of *N* = 20. We deemed this level of power to be sufficient to detect the main within-subjects effects of interest, though we note that between-group comparisons should be interpreted with caution due to low power for a between-subjects design. We therefore included 20 participants in each group.

### Stimuli

The experiment was presented using PsychoPy version 1.85.2 on a 15.4″ ProLite 39a screen by Ilyama (Group 1) or on a Samsung S22C450BW monitor with a 17″ screen diagonal (Group 2). The visuo-spatial stimuli which functioned as action effects consisted of three squares positioned horizontally across the screen. The squares were approximately 4 centimeters (cm) in height and width with a diagonal length of 5.5 cm, and they were spaced about 7 millimeters (mm) apart (in PsychoPy units, the size of the squares was set to “5 deg”, and spatial positions in *x*, *y* (horizontal, vertical) coordinates were set to − 6.0, 0.0, and 6.0). At the beginning of a trial, the three squares were presented with a black outline against a grey background (RGB: 128; 128; 128) and filled with the same color as the background (empty), first with a thin black outline of about one-third of a mm in width (line width of 1 in PsychoPy), and next with a thick black outline of about 1 mm in width (line width of 5 in PsychoPy). Subsequently, the outline of all squares was presented in one of four colors: (RGB) red (255; 1; 0), green (0; 123; 0), yellow (252; 248; 17), or blue (3; 1; 248). The color of the outline of the squares functioned as the action cue signifying the correct sequence of keypresses to be performed. The squares remained empty until the participant completed their response. After a correct response from the participant, each square then filled with the same color as the outline one at a time from right to left or left to right.

### Procedure

**Fig. 1:**
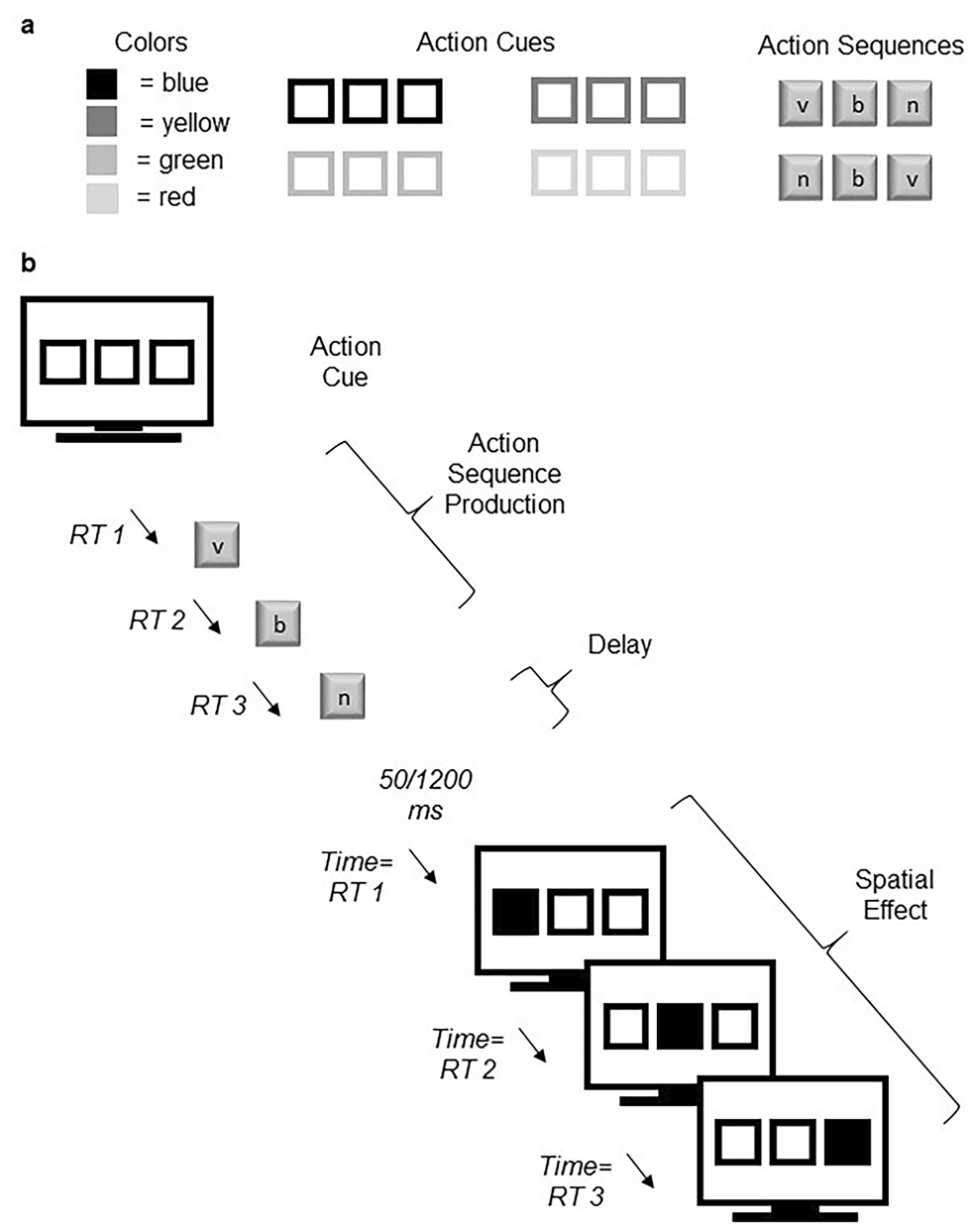
Task design. **a** The top panel shows which colors were used to cue each sequence. Note that the mapping of cue color to sequences was constant across participants (not counterbalanced). **b** The lower panel illustrates the progression of a trial within the task. Note that the timing with which the visual-spatial effects were presented was programmed to match the participant’s immediately-preceding production of the sequence, as follows: The time interval from the end of the delay to the onset of the first square being filled (the first action effect) was equivalent to the time interval between the onset of the color cue and the participant’s first keypress (“*RT 1*” above). The time interval between the first and second square being filled was equivalent to the time interval between the participant’s first and second keypress (“*RT 2*” above). The time interval between the second and third square being filled was equivalent to the time interval between the participant’s second and third keypress (“*RT 3*” above). The timing of the action effect stimuli therefore depended on the timing of the participant’s immediately preceding response. The last square to fill remained so for a fixed duration of 500 ms

Each trial in the experiment began with the presentation of three empty, horizontally arrayed squares with thin black outlines, presented for 1500 ms. The same squares with thick black outlines were then presented for 500 ms. The thick black squares signaled the beginning of a trial. The border of the squares was then presented in one of the four colors: red, green, blue, or yellow. The color was the cue that indicated the sequence that participants were to perform. Blue or yellow squares required participants to press the keys v, b, n (left-to-right). Green or red squares required participants to press the keys n, b, v (right-to-left) (see Fig. 1a). After the participant had responded with the correct sequence, a delay of 50 ms (short delay) or 1200 ms (long delay) occurred, after which the visuo-spatial action effects were presented. In the case of compatible visuo-spatial effects, the squares would fill with the same color as their outline successively, in the same direction as the performed sequence (left to right or right to left). In the case of incompatible action effects, the squares would fill in the reverse order as the performed sequence (for instance, action effects for the sequence v, b, n would be squares filling from right to left). Squares always filled with the outline color one at a time, such that when one square filled with the outline color, the previously-filled square would fill again with the background color. This created a dynamic visual effect: the filled squares appeared to move across the screen in either the same direction as the performed keypress sequence (spatially compatible action effects) or the reverse direction as the performed keypress sequence (spatially incompatible action effects) (see Fig. 1b). Note that here the visual effect always occurred after the sequence had been completed, in contrast to previous studies of effect anticipation in sequencing, in which one effect occurred after each response in the sequence (Keller & Koch, 2008). We manipulated the action effects in this manner to mimic the action–effect delay manipulation of previous studies, in which the delay was based on the interval from the offset of a single response to the onset of the effect (Dignath & Janczyk, 2017). Furthermore, the time at which each square appeared filled was programmed to mirror the timing of the participant’s immediately preceding sequence of keypresses (see Fig. 1 note for further explanation). When participants made an incorrect response, an error message reading “Falsch!” (German for “wrong!”) was presented for the same duration as the participant’s immediately preceding response (the time interval from the cue to the last keypress).

At the beginning of the experiment, participants first read a set of instructions that specified which sequence of keypresses to perform following which color cues. Participants next completed a practice block of 12 trials without any visuospatial action effects following the keypress responses, to practice retrieving the correct keypress sequence in response to the corresponding color cues. Stimulus color was entirely random during this practice block. Participants then completed four experimental blocks of 80 trials each (320 experimental trials total). Each block consisted of a combination of the spatial compatibility factor (compatible or incompatible action effect) and the delay factor (short or long delay between sequence production and action effect). For the remainder of the Method and Results sections we refer to these factors as Compatibility and Delay, respectively. Compatibility and Delay were fully crossed, resulting in four conditions: (1) compatible/short delay, (2) compatible/long delay, (3) incompatible/short delay, and (4) incompatible/long delay. These conditions were counterbalanced across participants such that levels of Compatibility were blocked (both compatible blocks were presented in either the first and second or third and fourth blocks), and levels of Delay always alternated between blocks (e.g., the short delay occurred in the first and third or second and fourth blocks). This resulted in four possible counterbalanced orders of the conditions (C = compatible, I = incompatible, s = short delay, l = long delay): Cs-Cl-Is-Il; Cl-Cs-Il-Is; Is-Il-Cs-Cl; or Il-Is-Cl-Cs. In addition, within each of the four experimental blocks, the color of the action cue varied pseudo-randomly from trial to trial. Because each keypress sequence was cued by two possible colors, there were three possible transitions between trials: (1) both the cue color and the sequence could repeat from one trial to the next, (2) both the cue color and the sequence could switch from one trial to the next, and (3) the cue color could switch while the sequence repeated from one trial to the next. For the remainder of the Method and Results sections, the factor signifying whether the cue or sequence switched or repeated across trials will be referred to as Transition. The above levels of Transition will be referred to as “Both Repeat” (cue and sequence repeat), “Both Switch” (cue and sequence switch), and “Cue Switch” (cue switches while sequence repeats), respectively. Each of the four cue colors was presented an equal number of times. The entire experiment lasted between 30 and 45 min.

In addition, the predictability of action sequence order across trials was varied between two groups of participants. All stimuli and procedures were identical between the two groups, with the exception that for Group 1, the order of correct keypress sequences was fixed across trials, and for Group 2 the order of correct keypress sequences was pseudorandom. For Group 1, the fixed pattern of required sequential responses followed an AABB pattern of two left-to-right (v-b-n) responses (blue or yellow cues) followed by two right-to-left (n-b-v) responses (red or green cues). For both groups, each of the four colors was presented an equal number of times. The number of cue/sequence switch and repetition trials in each condition was similar for both groups. For Group 1, the number of sequence switch and sequence repetition trials were nearly equal due to the fixed order of sequences (40 sequence repetitions and 39 sequence switches, omitting the first trial which could be neither a repeat or switch trial), and about half of the sequence repetition trials were also repetitions of the cue. The number of Cue Switch and Both Repeat trials was not identical due to the randomization of cue color across trials. For Group 2, the number of sequence switch and sequence repetition trials was similar in each condition. Because the order of sequences was randomized, approximately half of the trials were sequence repetitions, and of those trials approximately half were also cue repetitions. Thus, the percentage of Both Switch, Cue Switch, and Both Repeat trials in each condition (about 50%, 25%, and 25% respectively) was similar for Group 1 and Group 2. For the remainder of the Method and Results sections, the factor signifying the between-group variation in sequence predictability will be referred to as Group (Group 1: “Predictable Sequence Group”, Group 2: “Unpredictable Sequence Group”).

### Design and analyses

The design consisted of a 2 × 2 × 3 × 2 fixed factor structure with within-subjects factors Compatibility (compatible vs. incompatible), Delay (short vs. long), and Transition (Both Repeat, Both Switch, Cue Switch), and the between-subjects factor Group (Predictable Sequence vs. Unpredictable Sequence).

Three dependent variables were calculated. All data used to calculate the dependent variables excluded practice trials and the first trial of each experimental block. The first variable we refer to as “initiation time”, which was the time in milliseconds from the onset of the action cue (the color) to the onset of the first keypress in the sequence (in other words, the timestamp of the first keypress onset minus the timestamp of the cue onset). We interpret initiation time to include the time required to retrieve the correct action sequence and start execution. All trials in which initiation time differed from the initiation time mean (per Group) by more than three standard deviations were excluded from all further analysis. The second variable we refer to as “execution time”, which was the time in milliseconds from the onset of the first keypress of the sequence to the onset of the last keypress of the sequence (in other words, the timestamp of the final keypress minus the timestamp of the first keypress). We interpret this time interval to reflect the time required for motoric execution. All trials in which the times from the first to the second keypress and the second to the third keypress differed from their respective means by more than three standard deviations (per Group) were also excluded from further analysis. Both initiation and execution times additionally excluded all trials in which the sequence was performed incorrectly, and all trials immediately following incorrect sequence performance. The third variable, “error rate”, was the percentage of trials within a given experimental block in which the sequence was performed incorrectly. Error rate only included incorrect trials that followed a correctly-performed trial.

For each dependent variable (initiation time, execution time, and error rate), a mixed-effects ANOVA with a 2 × 2 × 3 × 2 fixed factor structure was run, each of which included the factors Compatibility (compatible vs. incompatible), Delay (short vs. long), Transition (Both Repeat vs. Both Switch vs. Cue Switch), and Group (Predictable Sequence vs. Unpredictable Sequence). A Greenhouse–Geisser sphericity correction was applied to the degrees of freedom for each *F* test involving the 3-level factor (Transition). The skewness and kurtosis values of the sampling distribution of each dependent variable in each condition fell within acceptable ranges (skewness *z* = [− 0.18, 1.4], kurtosis *z* = [− 0.1, 0.57]). Post-hoc pairwise comparisons between fixed factor levels included a Tukey adjustment. In addition to partial and generalized *η*^*2*^, for pairwise comparisons we report *Hedges’s g*_*rm*_ (for correlated samples) or *Hedges’s g*_*s*_ (for independent samples), which are variations of Cohen’s *d* that give a less biased estimation of population effect size (Lakens, 2013). Data processing and statistical analyses were performed in R version 4.0.1 using the afex and emmeans packages (Lenth, 2021; R Core Team, 2020; Singmann et al., 2021). Pairwise comparisons were conducted using the emmeans package (Lenth, 2021), which uses a pooled residual variance estimate and corresponding degrees of freedom, based on the estimated marginal means. Additionally, Online Appendix B reports pairwise *t* tests using the comparison-specific residual variance estimates to conduct the *t* tests.

We expected to replicate previously-documented effects of action–effect delay, even in the presence of spatial action effects, within the novel context of sequential action. We therefore predicted main effects of Delay such that initiation and execution of sequential actions would be faster for the short versus the long delay, regardless of Compatibility. We also expected main effects of Compatibility such that sequence initiation and execution would be faster and error rate lower for compatible compared to incompatible action effects. We also predicted effects of Transition (trial-to-trial repetition of the action cue or sequence), such that sequence initiation and execution would be faster and error rate lower for cue or sequence repetitions. In addition, we explored potential influences of cued action switches on Delay or Compatibility effects. We expected that switching could either reduce or increase the effects of Delay and Compatibility, which would be demonstrated by reduced or enhanced differences between compatible and incompatible conditions and between short and long delay in initiation time, execution time, and error rate, during switches compared to repeats. We expected Group to influence Transition effects, such that sequence order predictability would reduce the effects of cued action switches.

## Results

### Initiation time

Mean initiation times (in ms) per participant and condition were submitted to a 2 × 2 × 3 × 2 mixed-effects ANOVA. The ANOVA summary table with effect sizes is reported in Table 1, and post-hoc pairwise comparisons along with their associated marginal means, SEs, 95% confidence intervals, and effect sizes are reported in Table 2.

**Table 1:** ANOVA summary table for initiation time

Source	*df*	MSE	*F*	$\eta_{{\text{G}}}^{2}$	$\eta_{{\text{p}}}^{2}$	*p*
Delay	1, 38	9167.35	17.10***	0.013	0.31	< 0.001
Compatibility	1, 38	19,349.69	0.23	< 0.001	0.01	0.637
Transition	1.94, 73.69	5715.12	40.47***	0.038	0.52	< 0.001
Group	1, 38	246,356.36	3.15^+^	0.063	0.08	0.084
Delay × Group	1, 38	9167.35	4.71*	0.004	0.11	0.036
Compatibility × Group	1, 38	19,349.69	0.71	0.001	0.02	0.405
Transition × Group	1.94, 73.69	5715.12	3.80*	0.004	0.09	0.028
Compatibility × Delay	1, 38	7045.92	0.00	< 0.001	< 0.01	0.998
Compatibility × Transition	1.78, 67.53	1695.04	2.26	< 0.001	0.06	0.118
Delay × Transition	1.98, 75.35	1503.07	0.00	< 0.001	< 0.01	0.995
Group × Compatibility × Delay	1, 38	7045.92	0.05	< 0.001	< 0.01	0.818
Group × Compatibility × Transition	1.78, 67.53	1695.04	1.80	< 0.001	0.05	0.178
Group × Delay × Transition	1.98, 75.35	1503.07	1.08	< 0.001	0.03	0.346
Compatibility × Delay × Transition	1.96, 74.47	1727.69	1.14	< 0.001	0.03	0.323
Group × Compatibility × Delay × Transition	1.96, 74.47	1727.69	0.02	< 0.001	< 0.01	0.974

*MSE* mean square error

+*p* < 0.1. *p < 0.05. **p < 0.01. ***p < 0.001

**Table 2:** Post-hoc tests for initiation time

Delay									
Long			Short						
*M*	SE	95% CI	*M*	SE	95% CI	*df*	*t*	*p*	*g*_rm_
714	23.1	[667 760]	678	23.1	[631 724]	38	4.14***	< 0.001	0.24

Transition									
Both repeat			Both switch						
*M*	SE	95% CI	*M*	SE	95% CI	*df*	*t*	*p*	*g*_rm_
653	23.2	[607 700]	709	23.2	[662 756]	76	6.65***	< 0.001	0.38

Both repeat			Cue switch						
*M*	SE	95% CI	*M*	SE	95% CI	*df*	*t*	*p*	*g*_rm_
653	23.2	[607 700]	725	23.2	[678 772]	76	8.58***	< 0.001	0.49

Both switch			Cue switch						
*M*	SE	95% CI	*M*	SE	95% CI	*df*	*t*	*p*	*g*_rm_
709	23.2	[662 756]	725	23.2	[678 772]	76	1.93	0.138	0.11

Group									
Predictable			Unpredictable						
*M*	SE	95% CI	*M*	SE	95% CI	*df*	*t*	*p*	*g*_s_
655	32	[591 720]	736	32	[671 801]	38	1.78^+^	0.084	0.13

Delay × Group										
Group	Long			Short						
	*M*	SE	95% CI	*M*	SE	95% CI	*df*	*t*	*p*	*g*_rm_
Predictable	683	32.6	[617 749]	628	32.6	[562 694]	38	4.46*	< 0.001	0.26
Unpredictable	744	32.6	[679 810]	727	32.6	[661 793]	38	1.39	0.173	0.08

Transition × Group										
Transition	Predictable			Unpredictable						
	*M*	SE	95% CI	*M*	SE	95% CI	*df*	*t*	*p*	*g*_s_
Both Repeat	625	32.8	[559 691]	682	32.8	[615 748]	41.5	1.21	0.232	0.09
Both Switch	667	32.8	[601 733]	750	32.8	[684 816]	41.5	1.80^+^	0.080	0.13
Cue Switch	674	32.8	[608 740]	776	32.8	[710 842]	41.5	2.20*	0.033	0.15

*g*_*rm*_ Hedges’s *g*_rm_, *g*_*s*_ Hedges’s *g*_s_. Units =  milliseconds

^+^*p* < 0.1. *p < 0.05. **p < 0.01. ***p < 0.001

*Delay:* The ANOVA showed a main effect of Delay (see Table 1). Post-hoc pairwise comparisons showed that this main effect was due to shorter initiation times for the short delay compared to the long delay (see Table 2).

*Transition:* The ANOVA also showed a main effect of Transition on initiation time (see Table 1). Post-hoc pairwise comparisons showed shorter initiation times for Both Repeat transitions compared to Cue Switch transitions, as well as shorter initiation times for Both Repeat transitions compared to Both Switch transitions. No difference was detected between the Cue Switch and Both Switch conditions (see Table 2).

**Fig. 2:**
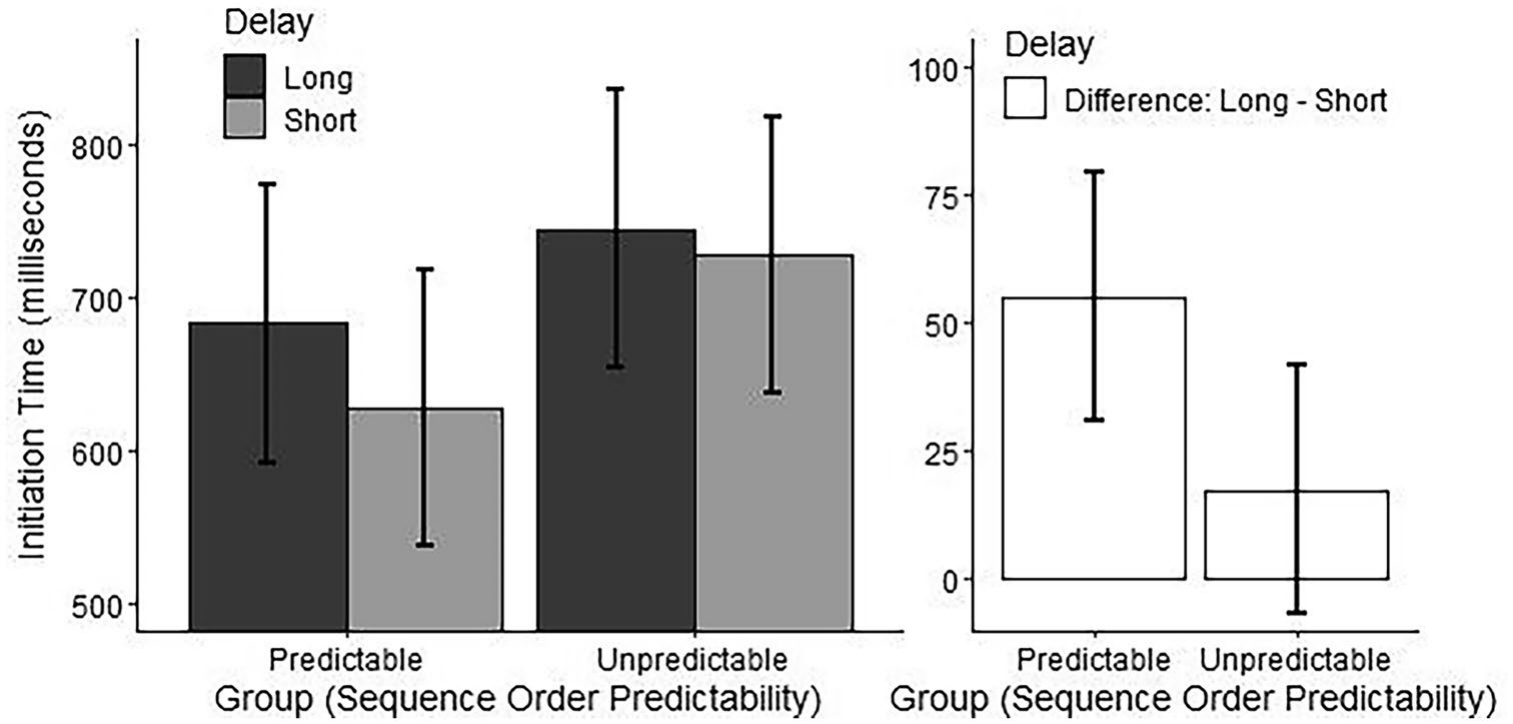
Effect of Delay by Group on sequence initiation time. Effect of Delay (Long = 1200 ms, Short = 50 ms) by Group (Predictable = Predictable Sequence Group with a fixed order of sequences across trials, Unpredictable = Unpredictable Sequence Group with a pseudorandom order of sequences across trials) on sequence initiation time. Marginal means are plotted in the left panel, and differences between the marginal means for levels of the factor Delay are plotted in the right panel. Error bars represent 95% confidence intervals

*Delay × Group:* The ANOVA also showed a Delay by Group interaction (see Table 1). Post-hoc pairwise comparisons showed shorter initiation time for the short delay compared to the long delay in the Predictable Sequence Group but no difference between the short and long delay in the Unpredictable Sequence Group (see Table 2 and Fig. 2).

*Transition × Group:* The ANOVA also showed a Transition by Group interaction (see Table 1). Post-hoc pairwise comparisons showed shorter initiation time in the Predictable Sequence Group compared to the Unpredictable Sequence Group in the Cue Switch condition, and a nearly-significant difference between groups in the Both Switch condition, but no difference between groups in the Both Repeat condition (see Table 2).

### Execution time

Mean execution times (ms) per participant and condition were submitted to a 2 × 2 × 3 × 2 mixed-effects ANOVA. The ANOVA summary table with effect sizes is reported in Table 3, and post-hoc pairwise comparisons along with their associated marginal means, SEs, and 95% confidence intervals, and effect sizes are reported in Table 4.

**Table 3:** ANOVA summary table for execution time

Source	*Df*	MSE	*F*	$\eta_{{\text{G}}}^{2}$	$\eta_{{\text{p}}}^{2}$	*p*
Delay	1, 38	3475.75	4.59*	0.007	0.11	0.039
Compatibility	1, 38	3324.04	0.07	< 0.001	< 0.01	0.793
Transition	1.43, 54.24	232.42	2.69^+^	< 0.001	0.07	0.093
Group	1, 38	50,662.54	0.00	< 0.001	< 0.01	0.987
Delay × Group	1, 38	3475.75	0.05	< 0.001	< 0.01	0.820
Compatibility × Group	1, 38	3324.04	0.11	< 0.001	< 0.01	0.739
Transition × Group	1.43, 54.24	232.42	1.34	< 0.001	0.03	0.265
Compatibility × Delay	1, 38	1157.65	3.23^+^	0.002	0.08	0.080
Compatibility × Transition	1.68, 63.92	80.28	0.32	< 0.001	0.01	0.688
Delay × Transition	1.77, 67.34	84.19	0.28	< 0.001	0.01	0.728
Group × Compatibility × Delay	1, 38	1157.65	2.01	0.001	0.05	0.164
Group × Compatibility × Transition	1.68, 63.92	80.28	1.27	< 0.001	0.03	0.283
Group × Delay × Transition	1.77, 67.34	84.19	0.59	< 0.001	0.02	0.537
Compatibility × Delay × Transition	1.58, 59.95	131.69	0.16	< 0.001	< 0.01	0.803
Group × Compatibility × Delay × Transition	1.58, 59.95	131.69	0.60	< 0.001	0.02	0.513

*MSE* mean square error

^+^*p* < 0.1. *p < 0.05. **p < 0.01. ***p < 0.001

**Table 4:** Post-hoc tests for execution time

Delay									
Long			Short						
*M*	SE	95% CI	*M*	SE	95% CI	*df*	*t*	*p*	*g*_rm_
419	10.6	[398 441]	408	10.6	[386 429]	38	2.14*	0.039	0.16

Transition									
Both repeat			Both switch						
*M*	SE	95% CI	*M*	SE	95% CI	*df*	*t*	*p*	*g*_rm_
413	10.3	[393 434]	415	10.3	[394 436]	76	1.24	0.431	0.03

Both repeat			Cue Switch						
*M*	SE	95% CI	*M*	SE	95% CI	*df*	*t*	*p*	*g*_rm_
413	10.3	[393 434]	412	10.3	[391 433]	76	1.07	0.533	0.02

Both switch			Cue switch						
*M*	SE	95% CI	*M*	SE	95% CI	*df*	*t*	*p*	*g*_rm_
415	10.3	[394 436]	412	10.3	[391 433]	76	2.32^+^	0.059	0.05

Delay × Compatibility										
Compatibility	Long			Short						
	*M*	SE	95% CI	*M*	SE	95% CI	*df*	*t*	*p*	*g*_rm_
Compatible	417	11.1	[395 439]	411	11.1	[389 433]	60.8	0.96	0.343	0.08
Incompatible	421	11.1	[399 444]	404	11.1	[382 427]	60.8	2.75**	0.008	0.24

*g*_*rm*_ Hedges’s *g*_rm_. Units =  milliseconds

^+^*p* < 0.1. *p < 0.05. **p < 0.01. ***p < 0.001

**Fig. 3:**
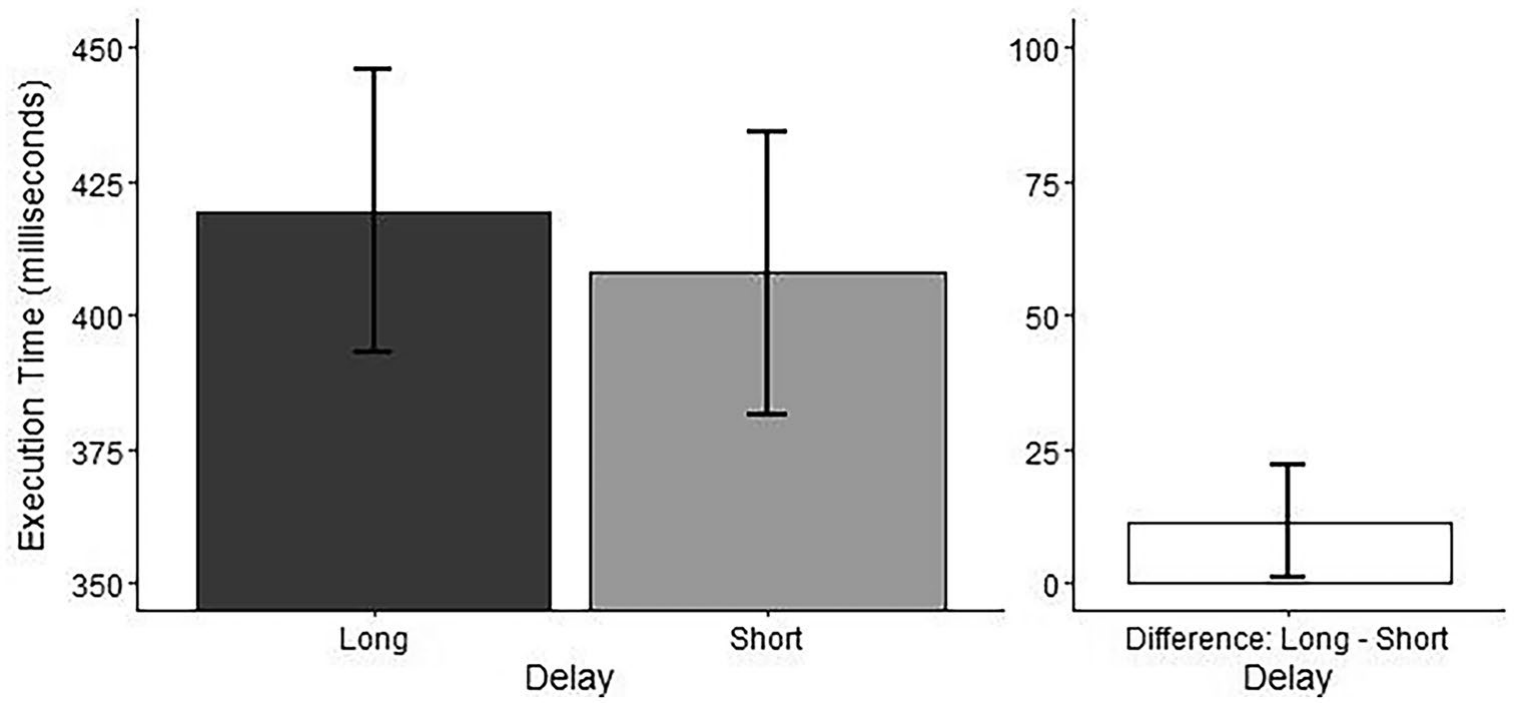
Effect of Delay on sequence execution time. Effect of Delay (Long = 1200 ms, Short = 50 ms) on sequence execution time. Marginal means are plotted in the left panel, and the difference between the marginal means for levels of the factor Delay are plotted in the right panel. Error bars represent 95% confidence intervals

*Delay:* The ANOVA showed a main effect of Delay (see Table 3). Post-hoc pairwise comparisons showed shorter execution times for the short delay compared to the long delay (see Table 4 and Fig. 3).

The ANOVA additionally showed a near-significant main effect of Transition and a near-significant Delay by Compatibility interaction (see Tables 3 and 4). While these results should be interpreted with caution, they suggested a possible trend toward longer execution time in the Both Switch condition compared to the Cue Switch condition, and they suggested a larger effect of Delay in the incompatible condition (shorter execution time for the short delay compared to the long delay in the incompatible condition but not the compatible condition).

*Supplementary analysis of RT2 and RT3*: We additionally examined participant’s second and third key presses (“RT2” and “RT3”, respectively) separately, to see whether the factors in the design had constant effects over the course of sequence execution (see Online Appendix A, Tables A1–A4). The results suggest that the delay anticipation effect on execution time persisted across the second and third responses of each action sequence, though the effect seems to be stronger for the second response.

*Supplementary analysis of change over each block of trials*: We also explored whether execution time changed at a different rate than initiation time over the course of each block of the experiment (see Online Appendix A, Table A5–A8). The results suggest that initiation time and execution time reduced over the course of the first block only, and that initiation time may have stabilized sooner than execution time.

*Supplementary analysis of execution time as a function of initiation time*: Regression analyses suggested that longer initiation time predicted longer execution time, both when Delay was short, and when Delay was long (see Online Appendix A, Tables A9 and A10).

### Error rate

Mean error rates (% incorrect trials) per participant and condition were submitted to a 2 × 2 × 3 × 2 mixed-effects ANOVA. The ANOVA summary table with effect sizes is reported in Table 5, and post-hoc pairwise comparisons along with their associated marginal means, SEs, and 95% confidence intervals, and effect sizes are reported in Table 6.

**Table 5:** ANOVA summary table for error rate

Source	*Df*	MSE	*F*	$\eta_{{\text{G}}}^{2}$	$\eta_{{\text{p}}}^{2}$	*p*
Delay	1, 38	11.64	0.16	< 0.001	< 0.01	0.691
Compatibility	1, 38	12.65	1.27	0.002	0.03	0.266
Transition	1.13, 42.81	55.19	9.61**	0.058	0.20	0.002
Group	1, 38	79.58	2.05	0.017	0.05	0.160
Delay × Group	1, 38	11.64	0.02	< 0.001	< 0.01	0.891
Compatibility × Group	1, 38	12.65	0.34	< 0.001	0.01	0.562
Transition × Group	1.13, 42.81	55.19	0.46	0.003	0.01	0.526
Compatibility × Delay	1, 38	8.05	0.66	< 0.001	0.02	0.422
Compatibility × Transition	1.50, 57.00	20.02	4.82*	0.015	0.11	0.019
Delay × Transition	1.72, 65.30	15.92	0.66	0.002	0.02	0.496
Group × Compatibility × Delay	1, 38	8.05	1.07	< 0.001	0.03	0.306
Group × Compatibility × Transition	1.50, 57.00	20.02	1.33	0.004	0.03	0.268
Group × Delay × Transition	1.72, 65.30	15.92	0.09	< 0.001	< 0.01	0.892
Compatibility × Delay × Transition	1.37, 51.92	17.72	0.43	0.001	0.01	0.578
Group × Compatibility × Delay × Transition	1.37, 51.92	17.72	0.19	< 0.001	0.01	0.746

*MSE* mean square error

^+^*p* < 0.1. *p < 0.05. **p < 0.01. ***p < 0.001

**Table 6:** Post-hoc tests for error rate

Transition									
Cue switch			Both repeat						
*M*	SE	95% CI	*M*	SE	95% CI	*df*	*t*	*p*	*g*_rm_
4.19	0.543	[3.11 5.27]	1.59	0.543	[0.51 2.67]	76	4.17***	< 0.001	0.75

Cue switch			Both switch						
*M*	SE	95% CI	*M*	SE	95% CI	*df*	*t*	*p*	*g*_rm_
4.19	0.543	[3.11 5.27]	2.15	0.543	[1.07 3.23]	76	3.27**	0.005	0.59

Both switch			Both repeat							
*M*	SE	95% CI	*M*	SE	95% CI		*df*	*t*	*p*	*g*_*rm*_
2.15	0.543	[1.07 3.23]	1.59	0.543	[0.51 2.67]		76	0.90	0.645	0.16

Transition	Transition × Compatibility									
	Compatible			Incompatible						
	*M*	SE	95% CI	*M*	SE	95% CI	*df*	*t*	*p*	*g*_rm_
Cue switch	5.09	0.62	[3.87 6.31]	3.29	0.62	[2.07 4.52]	113	3.01**	0.003	0.45
Both switch	2.24	0.62	[1.02 3.47]	2.06	0.62	[0.84 3.29]	113	0.30	0.762	0.05
Both repeat	1.15	0.62	[− 0.07 2.38]	2.03	0.62	[0.81 3.26]	113	1.47	0.144	0.22

*g*_*rm*_ Hedges’s *g*_rm_. Units =  milliseconds

^+^*p* < 0.1. *p < 0.05. **p < 0.01. ***p < 0.001

*Transition:* The ANOVA showed a main effect of Transition (see Table 5). Post-hoc pairwise comparisons showed higher error rate for Cue Switch transitions compared to Both Repeat transitions and Both Switch transitions, but no difference between the Both Repeat and Both Switch conditions (see Table 6).

**Fig. 4:**
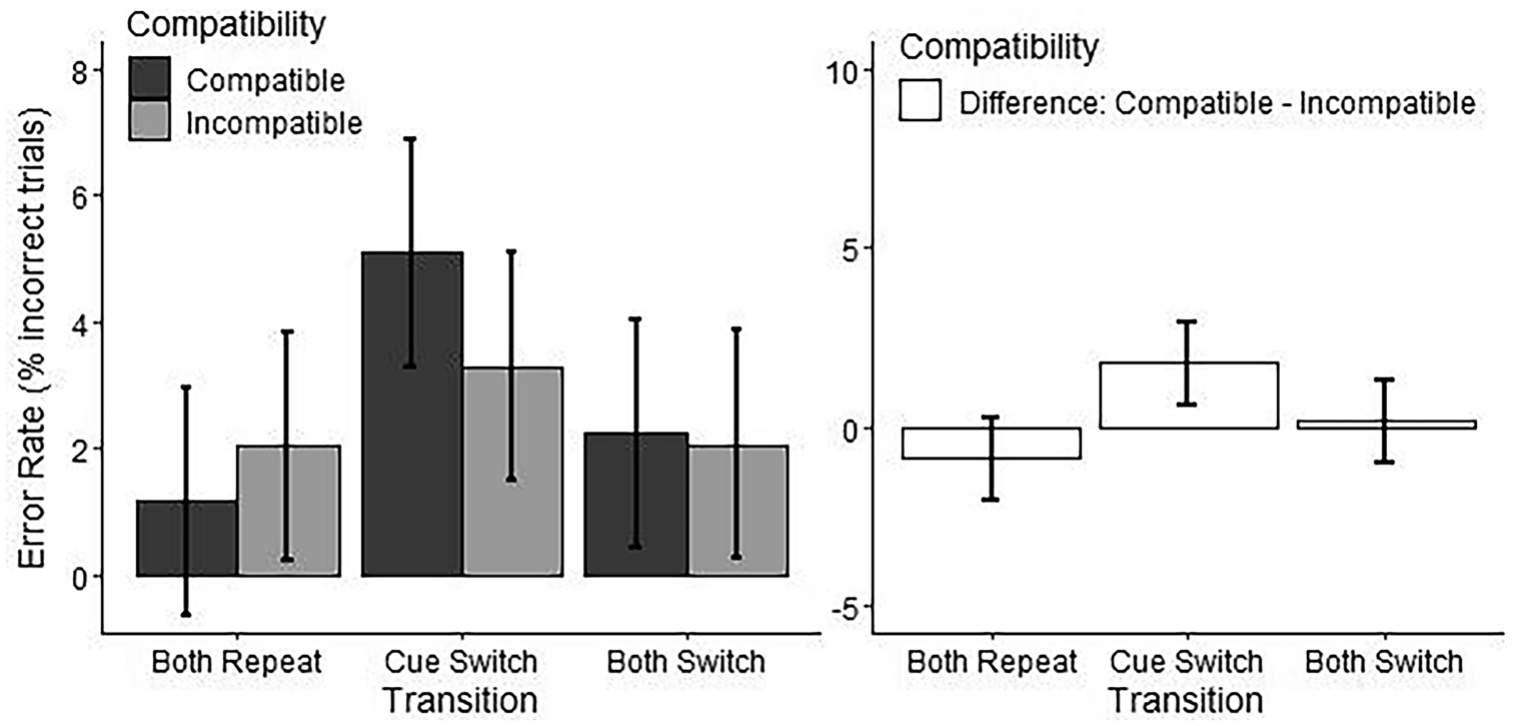
Effect of Transition by Compatibility on error rate. Effect of Transition (Both Repeat = cue and sequence repeat across trials, Cue Switch = cue switches while sequence repeats across trials, Both Switch = cue and sequence switch across trials) by Compatibility (Compatible = spatially compatible action effects, Incompatible = spatially incompatible action effects) on error rate. Marginal means are plotted in the left panel, and differences between the marginal means for levels of the factor Compatibility are plotted in the right panel. Error bars represent 95% confidence intervals

*Transition × Compatibility:* In addition, the ANOVA showed a Transition by Compatibility interaction (see Table 5). Post-hoc comparisons showed a greater error rate in the compatible condition compared to the incompatible condition for Cue Switch transitions, but no differences between the compatible and incompatible conditions for Both Switch or Both Repeat transitions (see Table 6 and Fig. 4).

To help interpret the interaction between Compatibility and Transition, a follow-up analysis was performed which examined whether increased errors when the cue switched in the compatible condition was due to retrieval of the incorrect sequence. We reasoned that a cue switch could have led participants to assume that the action sequence would also switch, and spatial compatibility could have facilitated retrieval of the incorrect sequence. To examine this possibility, errors were re-coded according to whether or not the performed sequence was a correct performance of the wrong sequence (the opposite sequence that should have been performed). Mean re-coded error rates per participant and condition were submitted to a 2 × 2 × 3 × 2 mixed-effects ANOVA. The ANOVA summary table with effect sizes is reported in Table 7, and post-hoc pairwise comparisons along with their associated marginal means, SEs, and 95% confidence intervals, and effect sizes are reported in Table 8. The ANOVA showed an interaction between Compatibility and Transition (see Table 7). Post-hoc pairwise comparisons showed a larger proportion of correctly-performed incorrect sequences for compatible compared to incompatible spatial effects in the Cue Switch condition (see Table 8). No differences between the compatible and incompatible conditions were detectable in the Both Switch condition or the Both Repeat condition (see Table 8). These results suggest that increased errors when the cue switched in the compatible condition may have been due to facilitated retrieval of the incorrect sequence.

**Table 7:** ANOVA summary table for re-coded error rate

Source	*df*	MSE	*F*	$\eta_{{\text{G}}}^{2}$	$\eta_{{\text{p}}}^{2}$	*p*
Delay	1, 38	6.56	2.04	0.003	0.05	0.162
Compatibility	1, 38	8.68	0.96	0.002	0.02	0.334
Transition	1.16, 43.98	23.01	18.75***	0.106	0.33	< 0.001
Group	1, 38	21.66	1.33	0.007	0.03	0.255
Delay × Group	1, 38	6.56	0.10	< 0.001	< 0.01	0.753
Compatibility × Group	1, 38	8.68	0.78	0.002	0.02	0.383
Transition × Group	1.16, 43.98	23.01	0.52	0.003	0.01	0.500
Compatibility × Delay	1, 38	5.62	0.24	< 0.001	0.01	0.626
Compatibility × Transition	1.31, 49.77	15.52	7.00**	0.033	0.16	0.006
Delay × Transition	1.33, 50.39	10.83	0.69	0.002	0.02	0.450
Group × Compatibility × Delay	1, 38	5.62	1.24	0.002	0.03	0.272
Group × Compatibility × Transition	1.31, 49.77	15.52	0.71	0.003	0.02	0.440
Group × Delay × Transition	1.33, 50.39	10.83	0.06	< 0.001	< 0.01	0.867
Compatibility × Delay × Transition	1.40, 53.18	5.18	0.25	< 0.001	0.01	0.703
Group × Compatibility × Delay × Transition	1.40, 53.18	5.18	1.19	0.002	0.03	0.299

*MSE* mean square error

^+^*p* < 0.1. *p < 0.05. **p < 0.01. ***p < 0.001

**Table 8:** Post-hoc tests for Transition × Compatibility, re-coded error rate

Transition	Transition × Compatibility									
	Compatible			Incompatible						
	*M*	SE	95% CI	*M*	SE	95% CI	*df*	*t*	*p*	*g*_rm_
Cue switch	3.57	0.40	[2.78 4.36]	1.78	0.40	[0.98 2.57]	113	3.65***	< 0.001	0.70
Both switch	0.40	0.40	[− 0.40 1.19]	0.74	0.40	[− 0.05 1.53]	113	0.69	0.500	0.13
Both repeat	0.12	0.40	[− 0.67 0.91]	0.79	0.40	[− 0.01 1.58]	113	1.35	0.180	0.26

*g*_*rm*_ Hedges’s *g*_rm_. Units = milliseconds

^+^*p* < .01. *p < 0.05. **p < 0.01. ***p < 0.001

## Discussion

We tested whether temporal and spatial features of action effects are retrieved and utilized in sequential action control, and whether temporal or spatial effect anticipation is influenced by higher-order planning (action switching across trials). We tested how sequential action control is affected by the delay between actions and effects (short versus long delay), spatial compatibility between actions and effects (compatible versus incompatible), and switching the action cue or the action sequence across trials with either a predictable or unpredictable order of sequences.

### Summary of main findings

The results replicate a delay-anticipation effect as demonstrated in previous studies, and the results extend this effect to sequential action control. A shorter delay between sequence production and effect onset speeded the time to both initiate and execute sequences, compared to a longer delay. The delay effect was modified by reduced higher-order planning demands: the effect on initiation times was stronger when action transitions were predictable (we discuss this result further below), even though it was about the same in execution times. The results additionally showed effects of cue/action switching. Speed and accuracy of sequence production were reduced most when only the cue switched, compared to when both the cue and action repeated, though sequence initiation also slowed when both the cue and action switched compared to when they both repeated. Post-hoc analyses suggested that cue switches activated the other action sequence, which was incorrect if the switched cue required repeating the previous action sequence. Finally, as expected, a fixed sequence order reduced slowing due to switching, particularly for the Cue Switch condition.

### Delay anticipation in sequential action

The current study extended the delay-anticipation effect to the planning and execution of sequential actions. This result supports the notion that effect anticipation can include anticipation of temporal features, such as the duration of the effect itself (Kiesel & Hoffmann, 2004; Kunde, 2003), or the temporal interval between the action and effect onset (Dignath & Janczyk, 2017; Dignath et al., 2014). These lines of work show that when action effects include longer temporal intervals, the time it takes to initiate the associated response also lengthens. It is assumed that longer temporal intervals take longer to retrieve, and thus slow response planning (Dignath & Janczyk, 2017). This assumption is supported by research on mentally simulated actions: A classical finding is that events that are longer or events that take longer to perform also take longer to imagine or simulate (Bart et al., 2020; Decety & Jeannerod, 1995; Jeannerod, 2001; Rieger et al., 2017). In addition, we here show that the delay-anticipation effect may be sensitive to higher-order action planning demands. The effect was weakened when the order of actions across trials was unpredictable, which likely increased the difficulty of switching between cued actions. It is possible that these switch demands overshadowed, rather than abolished, delay anticipation, as suggested by the large effect sizes of switch effects. It may be that participants found it easier to encode the temporal delays when switch demands were reduced, or temporal encoding may have been easier in a context of increased task predictability and reduced uncertainty. Because delay anticipation was not modified by the type of trial-to-trial transitions (whether the cue and/or action switched), the latter interpretation seems more plausible. This interpretation is consistent with previous studies showing that the success of temporal encoding depends to some extent on concurrent spatial features (Brown et al., 2013; Shin & Ivry, 2002; Thompson et al., 2001); for instance, temporal intervals between series of pitches were more poorly encoded when pitch was unpredictable (Bendixen et al., 2007). Temporal encoding may benefit from a predictable structure of both temporal and non-temporal events (Jones & Boltz, 1989).

We additionally show a delay-anticipation effect on sequence execution. This novel result suggests that temporal effect anticipation influences action control beyond the planning stage (see also Kunde et al., 2004). The influence of temporal anticipation on execution may reflect temporal adaptation. Various lines of evidence suggest that temporal regularities in the environment are encoded and retrieved for the control of attention (e.g., Correa et al., 2010; Jones & Boltz, 1989) and the control of action (e.g., Schroeder et al., 2010). For instance, organizing one’s movements in time (such as tapping along with a regular beat) is thought to involve the active retrieval (prediction) of the upcoming temporal interval (for a review see Repp, 2005). If participants in this study had successfully encoded and retrieved the temporal interval between their productions and the subsequent action effects, they may have adjusted their sequential movements in time accordingly.

It is important to note that a side-effect of the response-effect delay manipulation is that it also lengthens the overall timing of events across trials. A greater delay between response and effect also caused a larger delay between a response and the next stimulus (the next action cue), or a longer response-stimulus interval (RSI). RSI durations have been shown to influence response time, but importantly, in the opposite direction as that observed here. When RSIs are increased, response times reliably decrease (Klapp et al., 2019; Levy et al., 2006; McLeod, 1977; Rabbitt, 1969), presumably due to increased response preparation time. In the current study, we observed that the increased delay between response and the next stimulus lengthened response times rather than shortened them, as would be expected of a typical RSI effect. We therefore think it is unlikely that an RSI effect increased the difference between response times in the short-delay and long-delay conditions.

### Temporal and spatial anticipation in sequential action

The delay-anticipation effects reported here are consistent with the independent retrieval of temporal features, as suggested by the separate-trace account of effect anticipation (Dignath & Janczyk, 2017). Temporal effects influenced the time taken to initiate and execute action sequences, regardless of whether spatial effects were compatible or incompatible with sequential actions. These results suggest that temporal action effect features can be retrieved and utilized for action planning independently of spatial features of action effects. It is notable that visual-spatial compatibility had a weak-to-negligible effect on action control in the current study, unlike previous studies using auditory-spatial pitch effects in action sequences (Keller & Koch, 2008). We discuss the possible reasons behind the lack of spatial effects below (Limitations and future directions). Note however that the delay-anticipation effects reported here are similar to previous work, in which only one feature of action effects (temporal or spatial) tended to influence action control, even when multiple features were present (Dignath & Janczyk, 2017; Pfister et al., 2017). It is possible that when multiple features of action effects are present, only one feature dominates action control, as proposed previously (Dignath & Janczyk, 2017). Why this could be so remains to be explained. Further understanding may be gained by linking the separate trace account to other perspectives of action–effect integration. Action effect features may be processed independently because of constraints in action planning (Wirth et al., 2015), or the weighting of features based on their salience or utility in the task (Memelink & Hommel, 2013). Results from previous work seem to suggest the latter possibility. For instance, in a previous experiment that showed only temporal effect anticipation, response keys were mapped to non-temporal features in a more complex way than temporal effects (Dignath & Janczyk, 2017). In another study that only showed spatial effects, the difference between the short and long delay was relatively small (short delay = 300 ms, long delay = 400 ms) compared to other temporal manipulations (50 ms vs. 2000 ms or 50 ms vs. 1200 ms) (Pfister et al., 2017). Furthermore, previous observations of auditory pitch-based spatial action–effect compatibility point to a possible role of modality-specific processes in effect anticipation (Földes et al., 2017). Further research is needed to explain why action effect features may be retrieved independently, and under which circumstances.

### Action sequence switching

The current results further showed large effects of cued action sequence switching, modified by the predictability of action order from trial-to-trial. Sequence initiation was slowed most by switching only the cue, but also by switching both the cue and action, compared to repetition of both the cue and action. Error rate was also highest when only the cue switched, compared to when both the cue and action switched or repeated together. This decreased speed and accuracy following a switch could be understood as a classical “switch cost”, due to inhibition of current action parameters and re-retrieval of currently-inhibited action parameters, or it could be understood as a repetition benefit, due to the use of already-retrieved action parameters (Koch et al., 2018). The results here suggest a cost to switching a cued action, but most especially to switching only the action cue. The Cue Switch condition (a cue switch and action repetition) was more detrimental to speed and accuracy than the Both Switch condition (a cue and action switch). These results suggest that partially switching the action plan (just the cue) was generally more difficult and caused more errors than a complete action plan switch or repetition, even though a complete switch also slowed the initiation of the action. A cue switch may have “lured” participants into switching the action, either due to perceptual priming, or due to relatively easy action retrieval in this task (there were only two short sequential actions to remember). This “switch lure” could have overridden any potential action repetition benefit in this condition. This interpretation can also explain why errors further increased when spatial effects were action-compatible: spatially-compatible action effects may have further primed the alternate (wrong) sequence when the cue switched. We further suggest that cued action switching effects overshadowed spatial compatibility effects, which were not detected here. Finally, as expected, a fixed sequence order reduced slowing due to switching, particularly in the Cue Switch condition, suggesting that the fixed order reduced the relevance of the cues for signaling upcoming actions.

### Limitations and future directions

The spatial compatibility effects in the current study were small-to-negligible. This is a marked contrast to previous work showing robust spatial compatibility effects on action planning (Keller & Koch, 2008; Kunde, 2001). We believe the most likely explanation is that spatial compatibility effects were overshadowed by the demands of retrieving and switching between two cues per action sequence. The current task uniquely required participants to associate two cues per action. This requirement may have induced higher switching demands than in previous work. Cue switches were clearly the most difficult transitions in our task (see above). Our follow-up analysis on re-coded error rates showed that when the cue switched, spatial compatibility seemed to cause participants to perform the wrong sequence correctly, thus causing higher error rates. In addition, spatial compatibility effects may have been reduced by the manipulation. Action effects were presented after the entire sequence was performed: spatially-compatible action effects matched the sequence as a whole, such that a left-to-right sequence resulted in a left-to-right sequence of visual effects. This means, however, that the action effect immediately following the last response was incompatible: the right keypress was followed by a visual effect on the left. In other words, our manipulation created incompatibilities between specific responses and visual effects, within the globally-compatible response and effect. It is possible that this feature could have attenuated the perception of overall compatibility between actions and their effects. It is interesting to speculate whether the compatibility effect would take longer to develop over trials using our manipulation, because here it required participants to perceive the global or gestalt correspondence between the entire response and the entire sequence of visual effects, despite local action–effect incompatibilities. It could be interesting in future work to explore gestalt principles of perception in action–effect compatibility (see Klapp & Jagacinski, 2011).

The results additionally hinted at a possible interaction between action–effect delay and compatibility, during sequence execution. A delay-anticipation effect on sequence execution (faster execution for the short delay) was detectable only when the mapping between the sequence and spatial effects was incompatible, and not when the action–effect mapping was compatible. Although this effect is small and should be interpreted with caution, this pattern of results might not be consistent with a separate-trace account, which would predict reduced delay effects in the compatible condition due to overall speeding in the compatible condition (Dignath & Janczyk, 2017). Here, execution was not faster overall in the compatible condition, rather delay anticipation may have been amplified in the incompatible condition. This pattern could be consistent with feature-weighting (Memelink & Hommel, 2013): temporal features may have been more salient when spatial features were ambiguous. If the temporal-spatial interaction suggested here is reliable, this could suggest boundary conditions to the separate trace account, which could depend on the stage of action control (planning versus execution for instance). Or, as speculated above, the separate trace account may be a reflection of feature-weighting. These questions require further investigation.

We further note that in the current study the temporal and spatial action effects were not comparable in terms of their action-relevance. As discussed above, only the spatially compatible features were action-specific. Previous work compared features that were equally action-relevant, meaning that particular temporal and non-temporal features were mapped to specific responses (Dignath & Janczyk, 2017). Previous work on temporal action effects also mapped particular effect durations to particular responses (Kunde, 2003). Despite these differences in action-relevance, the current results suggest a consistency in delay-anticipation such that longer delays or durations slow action planning in either an action-specific or non-action-specific manner (Kunde, 2003). It would be interesting in further work to compare temporal and spatial action effects that are equally action-relevant.

It is interesting to consider how the temporal and spatial effect manipulations could have influenced action monitoring. In sequential actions such as typing, the visual effects may be used to monitor the correctness of performance. For instance, previous work showed that performance was slower when the visual effects of typing were eliminated, suggesting a compensatory strategy to enhance accuracy when visual feedback could not be used (Snyder et al., 2015). Monitoring may not be needed in all cases: studies of expert music performance suggest that eliminating auditory feedback does not alter performance fluency (Finney & Palmer, 2003; Repp, 1999). In the current study we suspect that the visual effects were not useful for monitoring action correctness: only spatially compatible effects could have been useful for monitoring, but these effects did not have a reliable influence on performance. However, a valuable direction for future work would be to examine the relationship between effect anticipation and action monitoring, particularly since both should rely on action–effect associations (e.g., Wolpert & Kawato, 1998; Ziessler et al., 2004).

Finally, it is interesting to note the relevance of effect anticipation to one’s sense of agency, or the subjective feeling of controlling the effects of one’s actions (Gallagher, 2000). Agency is thought to arise from the accurate prediction of sensory feedback from action (Frith et al., 2000), which results from action–effect binding in memory (Hommel et al., 2001; Miall & Wolpert, 1996). In accord with this view, participants report a reduced sense of agency when feedback is delayed, unpredictable, or spatially incompatible with respect to the preceding action (Couchman et al., 2012; Farrer et al., 2013; Krugwasser et al., 2019; Liesner et al., 2020). Some evidence suggests that temporal and spatial feedback manipulations affect agency separately (Sato & Yasuda, 2005), whereas other evidence suggests that they may have interactive effects on agency (Couchman et al., 2012). No studies to our knowledge have yet compared temporal manipulations with compatibility manipulations. Whether reduced agency in turn disrupts performance is uncertain (see Couchman et al., 2012) and is a fascinating topic for further work.

## Conclusions

The present findings show delay-anticipation effects in sequential action control, and thereby offer further support for the separate-trace account for multi-feature effect anticipation. Temporal action effects influenced the time taken to both initiate and execute sequential actions, independently of visual-spatial action effects. Delay-anticipation was sensitive to the predictability of upcoming actions, which may reflect an influence on task structure or coherence on encoding temporal features of action effects. The results also suggest a possible competitive interaction between temporal and spatial features, but this possibility needs further investigation. The results advance our understanding of multi-feature effect anticipation in action control, by extending the ideomotor principle and inviting additional perspectives to explain the complexity of everyday action.

## Supplementary Information

Below is the link to the electronic supplementary material.

Supplementary file1 (DOCX 126 kb)

## Data Availability

The datasets generated during and/or analysed during the current study are available at the following link: 10.23668/psycharchives.4861.

## Code availability

The code generated during the current study is available at the following link: 10.23668/psycharchives.4860.
