# Supplementary material for: The role of action effects in motor sequence planning and execution: exploring the influence of temporal and spatial effect anticipation
Source: Psychol Res. 2021 Jun 29;86(4):1078–96. doi: 10.1007/s00426-021-01525-2 (PMC9090704; doi:10.1007/s00426-021-01525-2)
Supplement: Supplementary file 1 — Supplementary file1 (DOCX 126 kb) [file 426_2021_1525_MOESM1_ESM.docx]

**Appendix A**

Supplementary Results

We performed additional analyses to examine participant’s second and third key presses (“RT2” and “RT3”, respectively) separately, to see whether the factors in the design had constant effects over the course of sequence execution. Mean RT2 and RT3 times (ms) per participant and condition were each submitted to a 2x2x3x2 mixed-effects ANOVA. The ANOVA summary table for RT2 is reported in Table A1, and post-hoc pairwise comparisons for RT2 are reported in Table A2. The ANOVA summary table for RT3 is reported in Table A3, and post-hoc pairwise comparisons for RT3 are reported in Table A4. The results suggest that the delay anticipation effect on execution time persisted across the second and third responses of each action sequence, though the effect seems to be stronger for the second response.

We also performed additional analyses to explore how initiation time and execution time changed over the course of each block of the experiment. We divided each block into 4 sections (which we refer to as “Block Section”) of 20 trials each. We then ran 2 separate 4 (Block Number) x 4 (Block Section) x 2 (Group) ANOVAs, one on initiation time (Tables A5 and A6) and one on execution time (Tables A7 and A8). We included Group as a factor in the ANOVAs because we thought it was possible for inter-trial predictability to influence adaptation over the course of the experiment blocks. The results are reported in Tables A5 – A8. The results can be summarized as follows: both initiation time and execution time only significantly reduced over the course of the first block in the experiment (as shown by an interaction between Block Number and Block Section in both ANOVAs). Post-hoc comparisons show that initiation time stabilized faster over the course of the first block: initiation time only decreased from section 1 to section 2 of the first block, with no further speeding. Execution time showed more gradual speeding: execution time reduced from section 1 to section 2, and from section 2 to section 3 of the first block. For the sake of simplicity only the post-hoc tests for the interactions between Block Section and Block Number are displayed in Tables A6 and A8, because these interactions most directly address the goal of these analyses (to examine adaptation over blocks).

We also examined whether execution time varied as a function of initiation time. We addressed this question using linear regression, with initiation time as the predictor variable and execution time as the predicted variable. These results are reported in Tables A9 and A10. We performed separate regressions for short and long delay conditions, each examining the relationship between mean initiation time and mean execution time across participants. As can be seen from the results, the initiation time was predictive of the execution time, in both the short and long delay conditions: longer initiation time predicted longer execution time.

**Table A1**

*ANOVA Summary Table for RT2*

| Source | *df* | MSE | *F* | *η^2^_G_* | *η^2^_p_* | *p* |  |
| --- | --- | --- | --- | --- | --- | --- | --- |
| Delay | 1, 38 | 818.15 | 5.15 * | .008 | .12 | .029 |  |
| Compatibility | 1, 38 | 684.06 | 0.20 | <.001 | <.01 | .659 |  |
| Transition | 1.58, 60.17 | 54.52 | 2.07 | <.001 | .05 | .145 |  |
| Group | 1, 38 | 11524.09 | 0.01 | <.001 | <.01 | .934 |  |
| Delay X Group | 1, 38 | 818.15 | 0.10 | <.001 | <.01 | .750 |  |
| Compatibility X Group | 1, 38 | 684.06 | 0.23 | <.001 | <.01 | .634 |  |
| Transition X Group | 1.58, 60.17 | 54.52 | 0.18 | <.001 | <.01 | .787 |  |
| Compatibility X Delay | 1, 38 | 260.85 | 2.40 | .001 | .06 | .130 |  |
| Compatibility X Transition | 1.70, 64.60 | 28.85 | 0.84 | <.001 | .02 | .418 |  |
| Delay X Transition | 1.63, 62.01 | 25.88 | 0.60 | <.001 | .02 | .518 |  |
| Group X Compatibility X Delay | 1, 38 | 260.85 | 0.89 | <.001 | .02 | .352 |  |
| Group X Compatibility X Transition | 1.70, 64.60 | 28.85 | 0.24 | <.001 | <.01 | .752 |  |
| Group X Delay X Transition | 1.63, 62.01 | 25.88 | 0.42 | <.001 | .01 | .620 |  |
| Compatibility X Delay X Transition | 1.67, 63.63 | 29.45 | 0.60 | <.001 | .02 | .525 |  |
| Group X Compatibility X Delay X Transition | 1.67, 63.63 | 29.45 | 0.05 | <.001 | <.01 | .929 |  |

*Note*. MSE = Mean square error. +p<.1. *p<.05. **p<.01. ***p<.001.

**Table A2**

*Post-hoc tests for RT2*

| Delay | | | | | |  |  |  |  |  |
| --- | --- | --- | --- | --- | --- | --- | --- | --- | --- | --- |
| Long | | | Short | | |  |  |  |  |  |
| *M* | *SE* | 95 % CI | *M* | *SE* | 95 % CI |  | *df* | *t* | *p* | *g_rm_* |
| 219 | 5.07 | [208 229] | 213 | 5.07 | [203 223] |  | 38 | 2.27 * | .029 | .19 |

*Note*. *g_rm_* = *Hedges’s g_rm._* Units = milliseconds. +p<.1. *p<.05. **p<.01. ***p<.001.

**Table A3**

*ANOVA Summary Table for RT3*

| Source | *df* | MSE | *F* | *η^2^_G_* | *η^2^_p_* | *p* |  |
| --- | --- | --- | --- | --- | --- | --- | --- |
| Delay | 1, 38 | 981.98 | 3.84 + | .006 | .09 | .058 |  |
| Compatibility | 1, 38 | 1143.72 | 0.01 | <.001 | <.01 | .915 |  |
| Transition | 1.47, 56.00 | 86.34 | 2.25 | <.001 | .06 | .129 |  |
| Group | 1, 38 | 14438.13 | 0.01 | <.001 | <.01 | .916 |  |
| Delay X Group | 1, 38 | 981.98 | 0.02 | <.001 | <.01 | .891 |  |
| Compatibility X Group | 1, 38 | 1143.72 | 0.04 | <.001 | <.01 | .842 |  |
| Transition X Group | 1.47, 56.00 | 86.34 | 2.42 | <.001 | .06 | .112 |  |
| Compatibility X Delay | 1, 38 | 351.57 | 3.72 + | .002 | .09 | .061 |  |
| Compatibility X Transition | 1.87, 71.21 | 49.08 | 1.39 | <.001 | .04 | .255 |  |
| Delay X Transition | 1.68, 63.86 | 46.04 | 0.63 | <.001 | .02 | .510 |  |
| Group X Compatibility X Delay | 1, 38 | 351.57 | 3.10 + | .002 | .08 | .086 |  |
| Group X Compatibility X Transition | 1.87, 71.21 | 49.08 | 1.23 | <.001 | .03 | .297 |  |
| Group X Delay X Transition | 1.68, 63.86 | 46.04 | 0.37 | <.001 | .01 | .655 |  |
| Compatibility X Delay X Transition | 1.59, 60.49 | 69.56 | 0.05 | <.001 | <.01 | .921 |  |
| Group X Compatibility X Delay X Transition | 1.59, 60.49 | 69.56 | 0.96 | <.001 | .02 | .370 |  |

*Note*. MSE = Mean square error. +p<.1. *p<.05. **p<.01. ***p<.001.

**Table A4**

*Post-hoc tests for RT3*

| Delay | | | | | |  |  |  |  |  |
| --- | --- | --- | --- | --- | --- | --- | --- | --- | --- | --- |
| Long | | | Short | | |  |  |  |  |  |
| *M* | *SE* | 95 % CI | *M* | *SE* | 95 % CI |  | *df* | *t* | *p* | *g_rm_* |
| 201 | 5.67 | [189 212] | 195 | 5.67 | [184 207] |  | 38 | 1.96 + | .058 | .17 |
| Delay X Compatibility | | | | | | |  |  |  |  |
| Compatibility | Long | | | Short | | |  |  |  |  |
|  | *M* | *SE* | 95 % CI | *M* | *SE* | 95 % CI | *df* | *t* | *p* | *g_rm_* |
| Compatible | 199 | 5.94 | [187 211] | 197 | 5.94 | [185 209] | 62.1 | 0.69 | .492 | .05 |
| Incompatible | 202 | 5.94 | [190 214] | 193 | 5.94 | [181 205] | 62.1 | 2.67 * | .010 | .24 |
| Delay X Compatibility X Group | | | | | | |  |  |  |  |
| Predictable | Long | | | Short | | |  |  |  |  |
|  | *M* | *SE* | 95 % CI | *M* | *SE* | 95 % CI | *df* | *t* | *p* | *g_rm_* |
| Compatible | 200 | 8.4 | [183 216] | 195 | 8.4 | [178 212] | 62.1 | 1.04 | .301 | .09 |
| Incompatible | 200 | 8.4 | [183 217] | 195 | 8.4 | [178 212] | 62.1 | 1.17 | .249 | .09 |
| Unpredictable | Long | | | Short | | |  |  |  |  |
|  | *M* | *SE* | 95 % CI | *M* | *SE* | 95 % CI | *df* | *t* | *p* | *g_rm_* |
| Compatible | 199 | 8.4 | [182 216] | 199 | 8.4 | [182 216] | 62.1 | 0.07 | .947 | .00 |
| Incompatible | 204 | 8.4 | [187 221] | 192 | 8.4 | [175 209] | 62.1 | 2.61 * | .011 | .22 |

*Note*. *g_rm_* = *Hedges’s g_rm._* Units = milliseconds. +p<.1. *p<.05. **p<.01. ***p<.001.

**Table A5**

*ANOVA Summary table for Initiation Time by Block Number, Block Section, and Group*

| Source | *df* | MSE | *F* | *η^2^_G_* | *η^2^_p_* | *p* |  |
| --- | --- | --- | --- | --- | --- | --- | --- |
| Block Section | 2.58, 97.98 | 4624.22 | 2.94 * | .002 | .07 | .044 |  |
| Block Number | 2.05, 77.88 | 17409.99 | 18.30 *** | .040 | .33 | <.001 |  |
| Group | 1, 38 | 335126.87 | 3.20 + | .064 | .08 | .082 |  |
| Block Section X Block Number | 6.56, 249.44 | 4861.41 | 2.25 * | .005 | .06 | .034 |  |
| Block Section X Group | 2.58, 97.98 | 4624.22 | 0.30 | <.001 | <.01 | .798 |  |
| Block Number X Group | 2.05, 77.88 | 17409.99 | 0.72 | .002 | .02 | .494 |  |
| Block Section X Block Number X Group | 6.56, 249.44 | 4861.41 | 1.68 | .003 | .04 | .120 |  |

*Note*. MSE = Mean square error. +p<.1. *p<.05. **p<.01. ***p<.001.

**Table A6**

*Post-hoc comparisons for Initiation Time, Block Section X Block Number*

|  |  | Block Section X Block Number | | | | | |  |  |  |  |
| --- | --- | --- | --- | --- | --- | --- | --- | --- | --- | --- | --- |
| Block Number | Block Section  Contrast | *M* | *SE* | 95 % CI | *M* | *SE* | 95 % CI | *df* | *t* | *p* | *g_rm_* |
| 1 | 1 - 2 | 793 | 25.5 | [742 844] | 739 | 25.5 | [688 790] | 455 | 4.06 *** | <.001 | .33 |
| 1 | 1 - 3 |  |  |  | 740 | 25.5 | [689 791] | 455 | 3.93 *** | <.001 | .33 |
| 1 | 1 - 4 |  |  |  | 740 | 25.5 | [689 791] | 455 | 3.93 *** | <.001 | .33 |
| 1 | 2 - 3 | 739 | 25.5 | [688 790] | 740 | 25.5 | [689 791] | 455 | -0.13 | .999 | .01 |
| 1 | 2 - 4 |  |  |  | 740 | 25.5 | [689 791] | 455 | -0.13 | .999 | .01 |
| 1 | 3 - 4 | 740 | 25.5 | [689 791] | 740 | 25.5 | [689 791] | 455 | -0.01 | 1.00 | .00 |
| 2 | 1 - 2 | 698 | 25.5 | [647 749] | 696 | 25.5 | [645 747] | 455 | 0.15 | .999 | .01 |
| 2 | 1 - 3 |  |  |  | 678 | 25.5 | [627 729] | 455 | 1.48 | .452 | .02 |
| 2 | 1 - 4 |  |  |  | 693 | 25.5 | [642 744] | 455 | 0.35 | .985 | .03 |
| 2 | 2 - 3 | 696 | 25.5 | [645 747] | 678 | 25.5 | [627 729] | 455 | 1.33 | .548 | .11 |
| 2 | 2 - 4 |  |  |  | 693 | 25.5 | [642 744] | 455 | 0.20 | .998 | .02 |
| 2 | 3 - 4 | 678 | 25.5 | [627 729] | 693 | 25.5 | [642 744] | 455 | -1.13 | .674 | .09 |
| 3 | 1 - 2 | 680 | 25.5 | [629 731] | 673 | 25.5 | [622 724] | 455 | 0.56 | .945 | .04 |
| 3 | 1 - 3 |  |  |  | 687 | 25.5 | [636 738] | 455 | -0.53 | .952 | .04 |
| 3 | 1 - 4 |  |  |  | 675 | 25.5 | [624 726] | 455 | 0.35 | .985 | .03 |
| 3 | 2 - 3 | 673 | 25.5 | [622 724] | 687 | 25.5 | [636 738] | 455 | -1.09 | .697 | .09 |
| 3 | 2 - 4 |  |  |  | 675 | 25.5 | [624 726] | 455 | -0.21 | .997 | .01 |
| 3 | 3 - 4 | 687 | 25.5 | [636 738] | 675 | 25.5 | [624 726] | 455 | .89 | .814 | .07 |
| 4 | 1 - 2 | 674 | 25.5 | [623 725] | 674 | 25.5 | [623 725] | 455 | 0.02 | 1.00 | .00 |
| 4 | 1 - 3 |  |  |  | 663 | 25.5 | [612 714] | 455 | 0.82 | .845 | .07 |
| 4 | 1 - 4 |  |  |  | 679 | 25.5 | [629 730] | 455 | -0.38 | .982 | .03 |
| 4 | 2 - 3 | 674 | 25.5 | [623 725] | 663 | 25.5 | [612 714] | 455 | 0.80 | .855 | .07 |
| 4 | 2 - 4 |  |  |  | 679 | 25.5 | [629 730] | 455 | -0.39 | .979 | .03 |
| 4 | 3 - 4 | 663 | 25.5 | [612 714] | 679 | 25.5 | [629 730] | 455 | -1.19 | .631 | .10 |

*Note*. *g_rm_* = *Hedges’s g_rm._* Units = milliseconds. +p<.1. *p<.05. **p<.01. ***p<.001.

**Table A7**

*ANOVA Summary table for Execution Time by Block Number, Block Section, and Group*

| Source | *df* | MSE | *F* | *η^2^_G_* | *η^2^_p_* | *p* |  |
| --- | --- | --- | --- | --- | --- | --- | --- |
| Block Section | 1.86, 70.61 | 1274.69 | 2.68 + | .002 | .07 | .080 |  |
| Block Number | 1.92, 72.80 | 3834.84 | 18.35 *** | .041 | .33 | <.001 |  |
| Group | 1, 38 | 67686.49 | 0.00 | <.001 | .00 | .955 |  |
| Block Section X Block Number | 4.94, 187.54 | 953.43 | 4.99 *** | .007 | .12 | <.001 |  |
| Block Section X Group | 1.86, 70.61 | 1274.69 | 0.13 | <.001 | <.01 | .865 |  |
| Block Number X Group | 1.92, 72.80 | 3834.84 | 2.30 | .005 | .06 | .110 |  |
| Block Section X Block Number  X Group | 4.94, 187.54 | 953.43 | 0.59 | <.001 | .02 | .703 |  |

*Note*. MSE = Mean square error. +p<.1. *p<.05. **p<.01. ***p<.001.

**Table A8**

*Post-hoc comparisons for Execution Time, Block Section X Block Number*

|  |  | Block Section X Block Number | | | | | |  |  |  |  |
| --- | --- | --- | --- | --- | --- | --- | --- | --- | --- | --- | --- |
| Block Number | Block Section  Contrast | *M* | *SE* | 95 % CI | *M* | *SE* | 95 % CI | *df* | *t* | *p* | *g_rm_* |
| 1 | 1 - 2 | 459 | 11.3 | [436 482] | 442 | 11.3 | [419 465] | 439 | 3.11 ** | .011 | .24 |
| 1 | 1 - 3 |  |  |  | 427 | 11.3 | [404 450] | 439 | 5.84 *** | <.001 | .44 |
| 1 | 1 - 4 |  |  |  | 426 | 11.3 | [403 449] | 439 | 6.01 *** | <.001 | .46 |
| 1 | 2 - 3 | 442 | 11.3 | [419 465] | 427 | 11.3 | [404 450] | 439 | 2.73 * | .034 | .21 |
| 1 | 2 - 4 |  |  |  | 426 | 11.3 | [403 449] | 439 | 2.90 * | .020 | .22 |
| 1 | 3 - 4 | 427 | 11.3 | [404 450] | 426 | 11.3 | [403 449] | 439 | 0.17 | .998 | .01 |
| 2 | 1 - 2 | 412 | 11.3 | [389 435] | 410 | 11.3 | [387 433] | 439 | 0.34 | .987 | .03 |
| 2 | 1 - 3 |  |  |  | 406 | 11.3 | [383 429] | 439 | 1.10 | .688 | .08 |
| 2 | 1 - 4 |  |  |  | 411 | 11.3 | [388 434] | 439 | 0.19 | .998 | .01 |
| 2 | 2 - 3 | 410 | 11.3 | [387 433] | 406 | 11.3 | [383 429] | 439 | 0.77 | .870 | .06 |
| 2 | 2 - 4 |  |  |  | 411 | 11.3 | [388 434] | 439 | -0.15 | .999 | .01 |
| 2 | 3 - 4 | 406 | 11.3 | [383 429] | 411 | 11.3 | [388 434] | 439 | -0.91 | .799 | .07 |
| 3 | 1 - 2 | 405 | 11.3 | [383 428] | 408 | 11.3 | [385 430] | 439 | -0.41 | .976 | .04 |
| 3 | 1 - 3 |  |  |  | 410 | 11.3 | [387 433] | 439 | -0.82 | .843 | .07 |
| 3 | 1 - 4 |  |  |  | 411 | 11.3 | [388 433] | 439 | -0.97 | .767 | .08 |
| 3 | 2 - 3 | 408 | 11.3 | [385 430] | 410 | 11.3 | [387 433] | 439 | -0.41 | .977 | .03 |
| 3 | 2 - 4 |  |  |  | 411 | 11.3 | [388 433] | 439 | -0.56 | .945 | .04 |
| 3 | 3 - 4 | 410 | 11.3 | [387 433] | 411 | 11.3 | [388 433] | 439 | -0.15 | .999 | .01 |
| 4 | 1 - 2 | 400 | 11.3 | [378 423] | 399 | 11.3 | [376 422] | 439 | 0.24 | .995 | .01 |
| 4 | 1 - 3 |  |  |  | 401 | 11.3 | [378 423] | 439 | -0.08 | .999 | .01 |
| 4 | 1 - 4 |  |  |  | 401 | 11.3 | [378 523] | 439 | -0.05 | 1.00 | .01 |
| 4 | 2 - 3 | 399 | 11.3 | [376 422] | 401 | 11.3 | [378 423] | 439 | -0.33 | .988 | .03 |
| 4 | 2 - 4 |  |  |  | 401 | 11.3 | [378 523] | 439 | -0.29 | .991 | .03 |
| 4 | 3 - 4 | 401 | 11.3 | [378 423] | 401 | 11.3 | [378 523] | 439 | 0.03 | 1.00 | .00 |

*Note*. *g_rm_* = *Hedges’s g_rm._* Units = milliseconds. +p<.1. *p<.05. **p<.01. ***p<.001.

**Table A9**

*Execution time as a function of Initiation time, Short Delay*

| Effect | *Estimate* | *SE* | *t* | *p* | *R^2^* | *Adjusted R^2^* |  |
| --- | --- | --- | --- | --- | --- | --- | --- |
| Intercept | 281.34 | 26.94 | 10.45 *** | <.001 |  |  |  |
| Initiation time | 0.19 | 0.04 | 4.83 *** | <.001 | .23 | .22 |  |

*Note*. +p<.1. *p<.05. **p<.01. ***p<.001.

**Table A10**

*Execution time as a function of Initiation time, Long Delay*

| Effect | *Estimate* | *SE* | *t* | *p* | *R^2^* | *Adjusted R^2^* |  |
| --- | --- | --- | --- | --- | --- | --- | --- |
| Intercept | 272.21 | 36.13 | 7.54 *** | <.001 |  |  |  |
| Initiation time | 0.21 | 0.05 | 4.19 *** | <.001 | .18 | .17 |  |

*Note*. +p<.1. *p<.05. **p<.01. ***p<.001.

**Appendix B**

Supplementary post-hoc pairwise comparisons

The following tables (Tables B1 to B8) report pairwise t-tests using the comparison-specific residual variance estimates. All reported *p* values are adjusted for multiple comparisons using the Tukey method.

**Table B1**

*Post-hoc tests for Initiation Time*

| Delay | | | | | |  |  |  |  |  |
| --- | --- | --- | --- | --- | --- | --- | --- | --- | --- | --- |
| Long | | | Short | | |  |  |  |  |  |
| *M* | *SE* | 95 % CI | *M* | *SE* | 95 % CI |  | *df* | *t* | *p* | *g_rm_* |
| 714 | 23.3 | [667 761] | 678 | 22.9 | [631 724] |  | 38 | 4.14 *** | <.001 | .24 |
| Transition | | | | | |  |  |  |  |  |
| Both Repeat | | | Both Switch | | |  |  |  |  |  |
| *M* | *SE* | 95 % CI | *M* | *SE* | 95 % CI |  | *df* | *t* | *p* | *g_rm_* |
| 653 | 21.2 | [611 696] | 709 | 23.9 | [660 757] |  | 38 | 6.53 *** | <.001 | .37 |
|  |  |  | Cue Switch | | |  |  |  |  |  |
|  |  |  | *M* | *SE* | 95 % CI |  | *df* | *t* | *p* | *g_rm_* |
|  |  |  | 725 | 24.3 | [676 774] |  | 38 | 8.06 *** | <.001 | .47 |
| Both Switch | | | Cue Switch | | |  |  |  |  |  |
| *M* | *SE* | 95 % CI | *M* | *SE* | 95 % CI |  | *df* | *t* | *p* | *g_rm_* |
| 709 | 23.9 | [660 757] | 725 | 24.3 | [676 774] |  | 38 | 2.12 | .100 | .10 |
| Group | | | | | |  |  |  |  |  |
| Predictable | | | Unpredictable | | |  |  |  |  |  |
| *M* | *SE* | 95 % CI | *M* | *SE* | 95 % CI |  | *df* | *t* | *p* | *g_s_* |
| 655 | 32 | [591 720] | 736 | 32 | [671 801] |  | 38 | 1.78 + | .084 | .13 |
| Delay X Group | | | | | | |  |  |  |  |
| Group | Long | | | Short | | |  |  |  |  |
|  | *M* | *SE* | 95 % CI | *M* | *SE* | 95 % CI | *df* | *t* | *p* | *g_rm_* |
| Predictable | 683 | 32.9 | [616 750] | 628 | 32.4 | [562 693] | 38 | 4.46 * | <.001 | .26 |
| Unpredictable | 744 | 32.9 | [678 811] | 727 | 32.4 | [662 793] | 38 | 1.39 | .514 | .08 |
| Transition X Group | | | | | | |  |  |  |  |
| Transition | Predictable | | | Unpredictable | | |  |  |  |  |
|  | *M* | *SE* | 95 % CI | *M* | *SE* | 95 % CI | *df* | *t* | *p* | *g_s_* |
| Both Repeat | 625 | 29.9 | [565 686] | 682 | 29.9 | [621 742] | 38 | 1.33 | .767 | .09 |
| Both Switch | 667 | 33.7 | [599 735] | 750 | 33.7 | [682 819] | 38 | 1.74 | .513 | .12 |
| Cue Switch | 674 | 34.4 | [604 744] | 776 | 34.4 | [706 845] | 38 | 2.10 | .312 | .14 |

*Note*. *g_rm_* = *Hedges’s g_rm._ g_s_* = *Hedges’s g_s_*. Units = milliseconds. +p<.1. *p<.05. **p<.01. ***p<.001.

**Table B2**

*Post-hoc tests for Execution Time*

| Delay | | | | | |  |  |  |  |  |
| --- | --- | --- | --- | --- | --- | --- | --- | --- | --- | --- |
| Long | | | Short | | |  |  |  |  |  |
| *M* | *SE* | 95 % CI | *M* | *SE* | 95 % CI |  | *df* | *t* | *p* | *g_rm_* |
| 419 | 11.82 | [395 443] | 408 | 9.26 | [389 427] |  | 38 | 2.14 * | .039 | .14 |
| Transition | | | | | |  |  |  |  |  |
| Both Repeat | | | Both Switch | | |  |  |  |  |  |
| *M* | *SE* | 95 % CI | *M* | *SE* | 95 % CI |  | *df* | *t* | *p* | *g_rm_* |
| 413 | 10.63 | [392 435] | 415 | 10.31 | [394 436] |  | 38 | 1.45 | .325 | .03 |
|  |  |  | Cue Switch | | |  |  |  |  |  |
|  |  |  | *M* | *SE* | 95 % CI |  | *df* | *t* | *p* | *g_rm_* |
|  |  |  | 412 | 9.97 | [392 432] |  | 38 | 0.84 | .680 | .01 |
| Both Switch | | | Cue Switch | | |  |  |  |  |  |
| *M* | *SE* | 95 % CI | *M* | *SE* | 95 % CI |  | *df* | *t* | *p* | *g_rm_* |
| 415 | 10.31 | [394 436] | 412 | 9.97 | [392 432] |  | 38 | 2.91 * | .016 | .04 |
| Delay X Compatibility | | | | | | |  |  |  |  |
| Compatibility | Long | | | Short | | |  |  |  |  |
|  | *M* | *SE* | 95 % CI | *M* | *SE* | 95 % CI | *df* | *t* | *p* | *g_rm_* |
| Compatible | 417 | 12.27 | [392 442] | 411 | 9.70 | [392 431] | 38 | 0.99 | .754 | .08 |
| Incompatible | 421 | 12.28 | [397 446] | 404 | 9.64 | [385 424] | 38 | 2.66 + | .053 | .22 |

*Note*. *g_rm_* = *Hedges’s g_rm._* Units = milliseconds. +p<.1. *p<.05. **p<.01. ***p<.001.

**Table B3**

*Post-hoc tests for Error Rate*

| Transition | | | | | |  |  |  |  |  |
| --- | --- | --- | --- | --- | --- | --- | --- | --- | --- | --- |
| Cue Switch | | | Both Repeat | | |  |  |  |  |  |
| *M* | *SE* | 95 % CI | *M* | *SE* | 95 % CI |  | *df* | *t* | *p* | *g_rm_* |
| 4.19 | 0.81 | [2.56 5.82] | 1.59 | 0.34 | [0.91 2.27] |  | 38 | 3.29 ** | .006 | .62 |
|  |  |  | Both Switch | | |  |  |  |  |  |
|  |  |  | *M* | *SE* | 95 % CI |  | *df* | *t* | *p* | *g_rm_* |
|  |  |  | 2.15 | 0.35 | [1.44 2.87] |  | 38 | 2.91 * | .016 | .45 |
| Both Switch | | | Both Repeat | | |  |  |  |  |  |
| *M* | *SE* | 95 % CI | *M* | *SE* | 95 % CI |  | *df* | *t* | *p* | *g_rm_* |
| 2.15 | 0.35 | [1.44 2.87] | 1.59 | 0.34 | [0.91 2.27] |  | 38 | 2.38 + | .057 | .25 |
|  | Transition X Compatibility | | | | | |  |  |  |  |
| Transition | Compatible | | | Incompatible | | |  |  |  |  |
|  | *M* | *SE* | 95 % CI | *M* | *SE* | 95 % CI | *df* | *t* | *p* | *g_rm_* |
| Cue Switch | 5.09 | 1.11 | [2.84 7.33] | 3.29 | 0.66 | [1.96 4.62] | 38 | 2.10 | .310 | .28 |
| Both Switch | 2.24 | 0.42 | [1.39 3.09] | 2.06 | 0.38 | [1.28 2.84] | 38 | 0.48 | .997 | .07 |
| Both Repeat | 1.15 | 0.31 | [0.52 1.79] | 2.03 | 0.47 | [1.07 2.99] | 38 | 2.00 | .359 | .33 |

*Note*. *g_rm_* = *Hedges’s g_rm._* Units = milliseconds. +p<.1. *p<.05. **p<.01. ***p<.001.

**Table B4**

*Post-hoc tests for Transition X Compatibility, Re-coded Error Rate*

|  | Transition X Compatibility | | | | | |  |  |  |  |
| --- | --- | --- | --- | --- | --- | --- | --- | --- | --- | --- |
| Transition | Compatible | | | Incompatible | | |  |  |  |  |
|  | *M* | *SE* | 95 % CI | *M* | *SE* | 95 % CI | *df* | *t* | *p* | *g_rm_* |
| Cue Switch | 3.57 | 0.87 | [1.82 5.32] | 1.78 | 0.28 | [1.21 2.34] | 38 | 2.34 | .205 | .38 |
| Both Switch | 0.40 | 0.15 | [0.10 0.69] | 0.74 | 0.20 | [0.32 1.15] | 38 | 1.36 | .749 | .30 |
| Both Repeat | 0.12 | 0.09 | [-0.05 0.30] | 0.79 | 0.26 | [0.26 1.31] | 38 | 2.44 | .168 | .53 |

*Note*. *g_rm_* = *Hedges’s g_rm._* Units = milliseconds. +p<.1. *p<.05. **p<.01. ***p<.001.

**Table B5**

*Post-hoc tests for RT2*

| Delay | | | | | |  |  |  |  |  |
| --- | --- | --- | --- | --- | --- | --- | --- | --- | --- | --- |
| Long | | | Short | | |  |  |  |  |  |
| *M* | *SE* | 95 % CI | *M* | *SE* | 95 % CI |  | *df* | *t* | *p* | *g_rm_* |
| 219 | 5.67 | [207 230] | 213 | 4.39 | [204 222] |  | 38 | 2.27 * | .029 | .16 |

*Note*. *g_rm_* = *Hedges’s g_rm._* Units = milliseconds. +p<.1. *p<.05. **p<.01. ***p<.001.

**Table B6**

*Post-hoc tests for RT3*

| Delay | | | | | |  |  |  |  |  |
| --- | --- | --- | --- | --- | --- | --- | --- | --- | --- | --- |
| Long | | | Short | | |  |  |  |  |  |
| *M* | *SE* | 95 % CI | *M* | *SE* | 95 % CI |  | *df* | *t* | *p* | *g_rm_* |
| 201 | 6.28 | [188 213] | 195 | 4.95 | [185 205] |  | 38 | 1.96 + | .058 | .15 |
| Delay X Compatibility | | | | | | |  |  |  |  |
| Compatibility | Long | | | Short | | |  |  |  |  |
|  | *M* | *SE* | 95 % CI | *M* | *SE* | 95 % CI | *df* | *t* | *p* | *g_rm_* |
| Compatible | 199 | 6.53 | [186 212] | 197 | 5.25 | [186 208] | 38 | 0.72 | .888 | .05 |
| Incompatible | 202 | 6.59 | [189 216] | 193 | 5.22 | [183 204] | 38 | 2.57 + | .065 | .22 |
| Delay X Compatibility X Group | | | | | | |  |  |  |  |
| Predictable | Long | | | Short | | |  |  |  |  |
|  | *M* | *SE* | 95 % CI | *M* | *SE* | 95 % CI | *df* | *t* | *p* | *g_rm_* |
| Compatible | 200 | 9.24 | [181 218] | 195 | 7.43 | [180 210] | 38 | 1.09 | .955 | .08 |
| Incompatible | 200 | 9.32 | [181 219] | 195 | 7.38 | [180 210] | 38 | 1.12 | .948 | .08 |
| Unpredictable | Long | | | Short | | |  |  |  |  |
|  | *M* | *SE* | 95 % CI | *M* | *SE* | 95 % CI | *df* | *t* | *p* | *g_rm_* |
| Compatible | 199 | 9.24 | [180 217] | 199 | 7.43 | [184 214] | 38 | 0.07 | 1.00 | .00 |
| Incompatible | 204 | 9.32 | [185 223] | 192 | 7.38 | [177 207] | 38 | 2.51 | .221 | .21 |

*Note*. *g_rm_* = *Hedges’s g_rm._* Units = milliseconds. +p<.1. *p<.05. **p<.01. ***p<.001.

**Table B7**

*Post-hoc comparisons for Initiation Time, Block Section X Block Number*

|  |  | Block Section X Block Number | | | | | |  |  |  |  |
| --- | --- | --- | --- | --- | --- | --- | --- | --- | --- | --- | --- |
| Block Number | Block Section  Contrast | *M* | *SE* | 95 % CI | *M* | *SE* | 95 % CI | *df* | *t* | *p* | *g_rm_* |
| 1 | 1 - 2 | 793 | 24.3 | [744 843] | 739 | 25.7 | [687 791] | 38 | 3.20 | .143 | .34 |
| 1 | 1 - 3 |  |  |  | 740 | 24.5 | [691 790] | 38 | 2.96 | .234 | .34 |
| 1 | 1 - 4 |  |  |  | 740 | 23.2 | [693 787] | 38 | 3.10 | .177 | .35 |
| 1 | 2 - 3 | 739 | 25.7 | [687 791] | 740 | 24.5 | [691 790] | 38 | -0.14 | 1.00 | .01 |
| 1 | 2 - 4 |  |  |  | 740 | 23.2 | [693 787] | 38 | -0.12 | 1.00 | .01 |
| 1 | 3 - 4 | 740 | 24.5 | [691 790] | 740 | 23.2 | [693 787] | 38 | -0.01 | 1.00 | .00 |
| 2 | 1 - 2 | 698 | 20.8 | [656 740] | 696 | 25.7 | [644 748] | 38 | 0.18 | 1.00 | .01 |
| 2 | 1 - 3 |  |  |  | 678 | 26.1 | [625 731] | 38 | 1.54 | .969 | .12 |
| 2 | 1 - 4 |  |  |  | 693 | 24.8 | [643 743] | 38 | 0.32 | 1.00 | .03 |
| 2 | 2 - 3 | 696 | 25.7 | [644 748] | 678 | 26.1 | [625 731] | 38 | 1.39 | .987 | .11 |
| 2 | 2 - 4 |  |  |  | 693 | 24.8 | [643 743] | 38 | 0.18 | 1.00 | .02 |
| 2 | 3 - 4 | 678 | 26.1 | [625 731] | 693 | 24.8 | [643 743] | 38 | -1.43 | .984 | .09 |
| 3 | 1 - 2 | 680 | 27.3 | [625 735] | 673 | 25.5 | [621 724] | 38 | 0.76 | 1.00 | .04 |
| 3 | 1 - 3 |  |  |  | 687 | 28.2 | [630 744] | 38 | -0.57 | 1.00 | .04 |
| 3 | 1 - 4 |  |  |  | 675 | 25.1 | [625 726] | 38 | 0.42 | 1.00 | .03 |
| 3 | 2 - 3 | 673 | 25.5 | [621 724] | 687 | 28.2 | [630 744] | 38 | -1.56 | .966 | .08 |
| 3 | 2 - 4 |  |  |  | 675 | 25.1 | [625 726] | 38 | -0.35 | 1.00 | .01 |
| 3 | 3 - 4 | 687 | 28.2 | [630 744] | 675 | 25.1 | [625 726] | 38 | 1.26 | .995 | .07 |
| 4 | 1 - 2 | 674 | 26.4 | [621 728] | 674 | 24.3 | [625 723] | 38 | 0.02 | 1.00 | .00 |
| 4 | 1 - 3 |  |  |  | 663 | 26.1 | [610 716] | 38 | 0.71 | 1.00 | .07 |
| 4 | 1 - 4 |  |  |  | 679 | 28.3 | [622 737] | 38 | -0.31 | 1.00 | .03 |
| 4 | 2 - 3 | 674 | 24.3 | [625 723] | 663 | 26.1 | [610 716] | 38 | 0.77 | 1.00 | .07 |
| 4 | 2 - 4 |  |  |  | 679 | 28.3 | [622 737] | 38 | -0.36 | 1.00 | .03 |
| 4 | 3 - 4 | 663 | 26.1 | [610 716] | 679 | 28.3 | [622 737] | 38 | -1.21 | .997 | .09 |

*Note*. *g_rm_* = *Hedges’s g_rm._* Units = milliseconds. +p<.1. *p<.05. **p<.01. ***p<.001.

**Table B8**

*Post-hoc comparisons for Execution Time, Block Section X Block Number*

|  |  | Block Section X Block Number | | | | | |  |  |  |  |
| --- | --- | --- | --- | --- | --- | --- | --- | --- | --- | --- | --- |
| Block Number | Block Section  Contrast | *M* | *SE* | 95 % CI | *M* | *SE* | 95 % CI | *df* | *t* | *p* | *g_rm_* |
| 1 | 1 - 2 | 459 | 11.9 | [435 483] | 442 | 14.0 | [414 470] | 38 | 2.10 | .755 | .20 |
| 1 | 1 - 3 |  |  |  | 427 | 14.6 | [398 457] | 38 | 3.56 + | .064 | .36 |
| 1 | 1 - 4 |  |  |  | 426 | 14.4 | [397 455] | 38 | 3.62 + | .055 | .38 |
| 1 | 2 - 3 | 442 | 14.0 | [414 470] | 427 | 14.6 | [398 457] | 38 | 3.48 + | .078 | .16 |
| 1 | 2 - 4 |  |  |  | 426 | 14.4 | [397 455] | 38 | 2.65 | .396 | .18 |
| 1 | 3 - 4 | 427 | 14.6 | [398 457] | 426 | 14.4 | [397 455] | 38 | 0.23 | 1.00 | .01 |
| 2 | 1 - 2 | 412 | 10.3 | [391 433] | 410 | 11.0 | [388 432] | 38 | 0.45 | 1.00 | .03 |
| 2 | 1 - 3 |  |  |  | 406 | 9.7 | [386 425] | 38 | 1.30 | .993 | .09 |
| 2 | 1 - 4 |  |  |  | 411 | 10.5 | [390 432] | 38 | 0.21 | 1.00 | .02 |
| 2 | 2 - 3 | 410 | 11.0 | [388 432] | 406 | 9.7 | [386 425] | 38 | 0.93 | 1.00 | .06 |
| 2 | 2 - 4 |  |  |  | 411 | 10.5 | [390 432] | 38 | -0.20 | 1.00 | .01 |
| 2 | 3 - 4 | 406 | 9.7 | [386 425] | 411 | 10.5 | [390 432] | 38 | -1.19 | .997 | .08 |
| 3 | 1 - 2 | 405 | 10.7 | [384 427] | 408 | 11.4 | [385 431] | 38 | -0.65 | 1.00 | .04 |
| 3 | 1 - 3 |  |  |  | 410 | 12.3 | [385 435] | 38 | -0.94 | 1.00 | .06 |
| 3 | 1 - 4 |  |  |  | 411 | 11.4 | [388 434] | 38 | -0.97 | .999 | .08 |
| 3 | 2 - 3 | 408 | 11.4 | [385 431] | 410 | 12.3 | [385 435] | 38 | -0.43 | 1.00 | .03 |
| 3 | 2 - 4 |  |  |  | 411 | 11.4 | [388 434] | 38 | -0.51 | 1.00 | .04 |
| 3 | 3 - 4 | 410 | 12.3 | [385 435] | 411 | 11.4 | [388 434] | 38 | -0.21 | 1.00 | .01 |
| 4 | 1 - 2 | 400 | 9.7 | [381 420] | 399 | 7.8 | [383 415] | 38 | 0.38 | 1.00 | .02 |
| 4 | 1 - 3 |  |  |  | 401 | 9.7 | [381 420] | 38 | -0.08 | 1.00 | .02 |
| 4 | 1 - 4 |  |  |  | 401 | 9.7 | [381 420] | 38 | -0.04 | 1.00 | .02 |
| 4 | 2 - 3 | 399 | 7.8 | [383 415] | 401 | 9.7 | [381 420] | 38 | -0.34 | 1.00 | .03 |
| 4 | 2 - 4 |  |  |  | 401 | 9.7 | [381 420] | 38 | -0.29 | 1.00 | .03 |
| 4 | 3 - 4 | 401 | 9.7 | [381 420] | 401 | 9.7 | [381 420] | 38 | 0.06 | 1.00 | .00 |

*Note*. *g_rm_* = *Hedges’s g_rm._* Units = milliseconds. +p<.1. *p<.05. **p<.01. ***p<.001.
